# Prey Selection by an Apex Predator: The Importance of Sampling Uncertainty

**DOI:** 10.1371/journal.pone.0047894

**Published:** 2012-10-26

**Authors:** Miranda L. Davis, Philip A. Stephens, Stephen G. Willis, Elena Bassi, Andrea Marcon, Emanuela Donaggio, Claudia Capitani, Marco Apollonio

**Affiliations:** 1 School of Biological and Biomedical Sciences, University of Durham, Durham, County Durham, United Kingdom; 2 Department of Natural and Environmental Sciences, University of Sassari, Sassari, Sardinia, Italy; California State University Fullerton, United States of America

## Abstract

The impact of predation on prey populations has long been a focus of ecologists, but a firm understanding of the factors influencing prey selection, a key predictor of that impact, remains elusive. High levels of variability observed in prey selection may reflect true differences in the ecology of different communities but might also reflect a failure to deal adequately with uncertainties in the underlying data. Indeed, our review showed that less than 10% of studies of European wolf predation accounted for sampling uncertainty. Here, we relate annual variability in wolf diet to prey availability and examine temporal patterns in prey selection; in particular, we identify how considering uncertainty alters conclusions regarding prey selection.

Over nine years, we collected 1,974 wolf scats and conducted drive censuses of ungulates in Alpe di Catenaia, Italy. We bootstrapped scat and census data within years to construct confidence intervals around estimates of prey use, availability and selection. Wolf diet was dominated by boar (61.5±3.90 [SE] % of biomass eaten) and roe deer (33.7±3.61%). Temporal patterns of prey densities revealed that the proportion of roe deer in wolf diet peaked when boar densities were low, not when roe deer densities were highest. Considering only the two dominant prey types, Manly's standardized selection index using all data across years indicated selection for boar (mean = 0.73±0.023). However, sampling error resulted in wide confidence intervals around estimates of prey selection. Thus, despite considerable variation in yearly estimates, confidence intervals for all years overlapped. Failing to consider such uncertainty could lead erroneously to the assumption of differences in prey selection among years. This study highlights the importance of considering temporal variation in relative prey availability and accounting for sampling uncertainty when interpreting the results of dietary studies.

## Introduction

Predator populations that have long been subjected to persecution are receiving increased conservation attention and are recovering in both North America and Europe [1]–[3]. Predicting the impact of changing predator numbers on prey species is important for managing populations of both predators and their prey [4]–[6]. Accurate predictions require a thorough understanding of predator diets and prey selection, which can be affected by a multitude of factors including: prey and predator densities [7]; the functional and numerical responses of predators to changes in prey density [8]–[9]; community composition (particularly the presence of alternative prey [10]–[11]); climatic conditions [12]; vegetation productivity [13]–[14]; and landscape heterogeneity [15]. These drivers can result in considerable temporal and spatial variation in patterns of predation. For this reason, studies of predation often require large sample sizes and high quality data to overcome uncertainty. However, because large predators are generally elusive and exist at low densities, they are expensive and time-consuming to study, meaning that large sample sizes are rare and results must usually be interpreted with caution. Failure to describe adequately the uncertainty in a dataset can promote misleading conclusions about predator feeding habits.

In Europe, the wolf (*Canis lupus*) is recovering from centuries of persecution. The expansion of wolf populations in many European countries [3] has the potential to change fundamentally the ecology of communities by exposing large ungulates to natural predation after decades (and in some cases, centuries) of predator absence. In North America, wolves limit ungulates in some areas [8], [16] and predation by recovering wolf populations has triggered complex trophic cascades, altering prey distribution and plant recruitment [2], [17]. Studies of ungulate dynamics and distributions in Europe indirectly suggest that wolves might play a similar role by limiting prey [11], [14], [18] but the intricacies of wolf-prey relationships and the potential for trophic cascades in European communities is poorly understood [19]–[20]. Dietary studies that accurately describe wolf prey selection are a necessary first step toward understanding wolf predation impacts on European wildlife.

Over the past three decades, scat analysis has been used to describe the dietary composition and prey selection of wolves, and to estimate their potential impact on prey communities [18], [21]–[27]. Scat-based dietary studies in Europe have highlighted the flexibility of the wolf as a predator. This variability is especially evident from reports of wild boar (*Sus scrofa*) utilisation among sites. Based on a review of results from the Bialowieza Primeval Forest (BPF), Poland, and other literature, Okarma [11] concluded that wild boar are generally avoided, while red deer (*Cervus elaphus*) are the prey of choice. However, BPF has a diverse ungulate community comprising 5 species (*Cervus elaphus*, *Sus scrofa*, *Capreolus capreolus*, *Alces alces*, *Bison bonasus*), some of which are no longer common elsewhere in modern-day Europe. By contrast, studies in southern and Mediterranean areas of Europe indicate that boar are sometimes preferred as prey [22]–[23], [25], [28]–[29]. Some of these southern sites are dominated by only two species, wild boar and roe deer (*Capreolus capreolus*), and could be considered more representative of communities throughout much of Europe [30]. Selection between these two prey appears to vary both among and within sites. This has been attributed to a variety factors including differences in community composition and in the vulnerability of individuals (as influenced by age, body size, grouping behaviour and season); unfortunately, the data required to distinguish between these alternatives are lacking [23], [25], [28], [31].

Some of the apparent variability in wolf diet may be a result of the scat analysis methods that are widely used to determine diet. Several papers have highlighted potential pitfalls in the scat analysis process, including those which may arise from the analysis of small datasets [32]–[36]. The potential for sampling error to arise is particularly high when the number of scats collected is small relative to the number produced by the study population. Such samples might not be representative and can lead to incorrect conclusions about diet, especially when the uncertainty in estimates based on small samples is not reported. Reynolds and Aebischer [33] advocated the use of re-sampling techniques (e.g. bootstrapping) to produce confidence intervals around estimates of dietary composition. While some recent studies (e.g. [35]) have used re-sampling techniques, much of the existing literature on European wolf diet does not account for uncertainty due to sampling error in results (20 out of 22 studies examined; Text S1, Table S1). In addition, studies of prey selection require estimates of prey availability, which are themselves subject to error. Failure to consider uncertainty in both prey use and prey availability can result in inappropriate conclusions.

Predation patterns may be further obscured by neglecting variation in prey selection among years, within a site. Many studies of wolf diet are either relatively short or pool scat samples across years (to increase sample size), thereby obscuring inter-annual variation (Text S1, Table S1). Mattioli et al. [29] found that prey use can vary substantially among years and that much of this variation is unaccounted for by the changing abundance of prey. Environmental factors affecting prey vulnerability (e.g. weather conditions, land use) may vary substantially from one year to the next, creating variability that could underlie some of the inconsistencies observed in wolf predation among sites. Long term studies that explicitly incorporate this variability will facilitate comparisons of wolf diet among sites and enable the identification of potential drivers of predation patterns across the continent.

In this study, we combine re-sampling techniques with nine years' scat sampling and drive census data to address the following questions regarding the dietary habits of wolves in Alpe di Catenaia: 1) do the wolves select for either of the two main prey species available, roe deer and wild boar? 2) how might an explicit consideration of uncertainty affect our conclusions about wolf dietary selection? and 3) how does wolf diet relate to the relative availability of prey species in the area?

## Methods

### Study area

**Figure 1:**
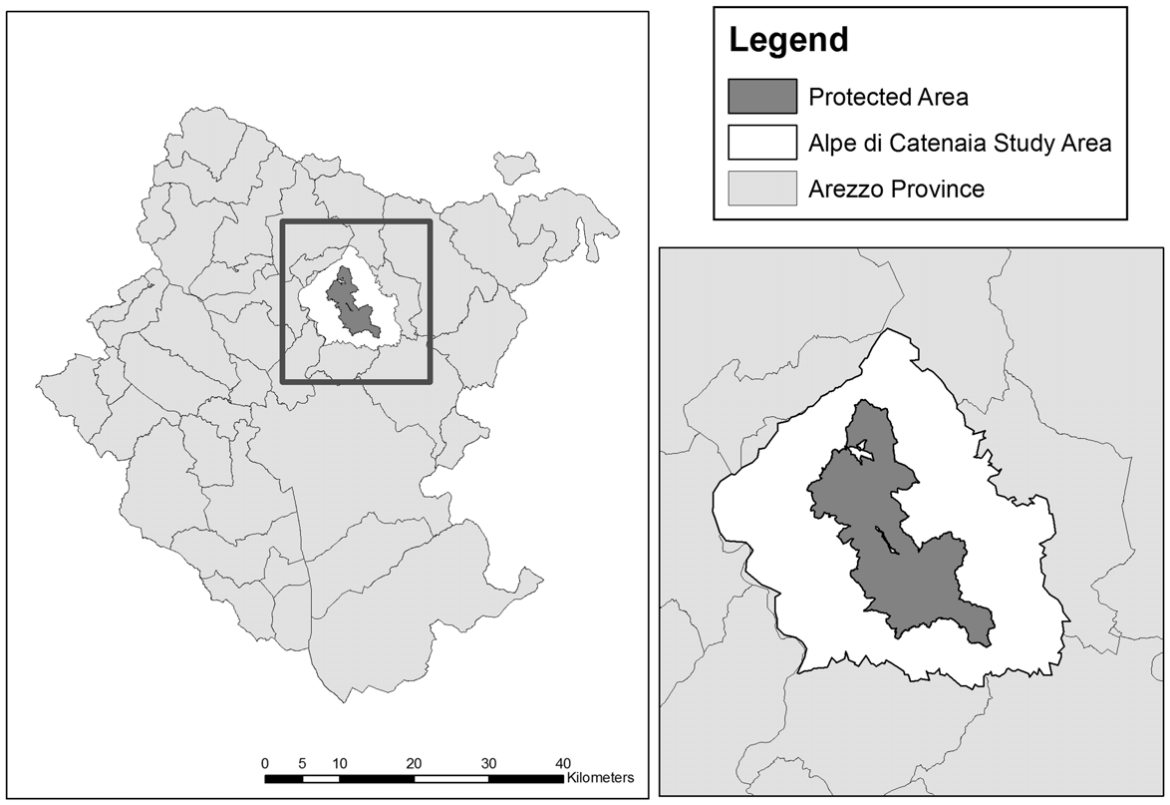
Alpe di Catenaia, Italy. The Alpe di Catenaia study site is located in the Arezzo province in Northern Tuscany, Italy. The study site includes a central protected area, where hunting is prohibited.

The 120 km^2^ Alpe di Catenaia study area is in the Apennine mountains in the north-east of Tuscany, Italy (Arezzo province, 43°48′N, 11°49′E). A 27 km^2^ area within this site is a protected area where hunting is banned (Fig. 1). Altitude within Alpe di Catenaia ranges from 300 to 1414 m above sea level. Vegetation cover is mainly composed of mixed deciduous hardwoods (76% of total area), dominated by oak (Quercus *spp.*), chestnut (*Castanea sativa*) and beech (*Fagus sylvatica*). The climate is temperate and seasonal with hot, dry summers, and cold, wet winters. Snowfall usually starts in October and may continue through April. There are a number of farms surrounding the study area which raise livestock (mostly sheep) that are a potential additional source of prey for wolves.

### Prey density and biomass estimation

The wild ungulate community included only wild boar and roe deer for the first seven years of the study; red deer have been occasionally recorded in the study area since 2007. Densities of wild boar and roe deer were estimated from drive censuses completed every May (2000–2005, and 2007–2008; method also described by Mattioli et al. [28]) by the Provincial Administration of Arezzo; the 2006 census excluded a large portion of the study area, so was excluded from our analyses. Census work was undertaken following accepted guidelines for monitoring wild ungulates, with permission granted by the Regional Government of Tuscany and Provincial Government of Arezzo. Censuses took place in both the protected and non-protected parts of the study area each year, encompassing about 80% wooded area and 20% other cover types. Government employees, researchers, and volunteers encircled an area of forest (each 0.14–0.52 km^2^ in size) then moved inwards and counted wild boar and roe deer observed in the contained area. Between 9 and 15 such forest blocks were sampled each year. The average density of observers during these surveys was approximately 110 persons per km^2^ [24]. In order to extrapolate from the surveyed areas to estimates of overall density at the site, we corrected for the differences in block area and the forest cover surrounding each block. The latter is necessary because wooded areas surrounded by more open habitat could appear to have higher densities of animals because during drives animals congregate in the more sheltered, forested areas [37]. The percentage area covered by forest within a 1 km buffer surrounding each forest block was extracted using GIS (ArcGIS version 10 [38]). The corrected density of animals within each surveyed block was thus calculated as number of individuals counted divided by block area and multiplied by percentage forest cover of the surrounding area (median value 81%, range 41–96.1% across blocks). The overall density of wild boar and roe deer at the site was then estimated as the mean across the different blocks. Drive census are a widely used technique and, while some animals are not seen during a census, it has been found that such drive census generally give higher density estimates than alternative methods [39]. To convert densities to biomass densities (kg per km^2^) we used the average body mass of boar (43.2±0.33 [SE] kg, n = 5003) and roe deer (21.1±0.12 [SE] kg, n = 2355) hunted in the districts that immediately surround the protected area (all age classes included).

### Scat collection and assessment of wolf diet

During the study period the area supported a single wolf pack which contained 3–6 individuals. This was confirmed using genetic analysis of scats (unpublished data), snow-tracking [18] and wolf-howling surveys [40]. Similar to the drive censuses, this work was undertaken with permission from the governments of Arezzo and Tuscany. Wolf scats were collected monthly between May 2000 and April 2009 from seven transects distributed throughout the study area (total length: 73 km per month). Years were defined as extending from May to the following April (i.e. scats collected between May 2000 and April 2001 were assigned to the year 2000–01). Scats were washed and the recovered prey remains were oven-dried at 68°C for 24 hours. Prey categories included wild boar, roe deer, red deer, hare (*Lepus europaeus*), small rodents, goats, sheep and cattle. Prey remains were identified through comparison to a reference collection of mammal hair, bones, and teeth collected from within the study area. Specimens were identified to species and age-class (for ungulates only) when possible. This identification was based on the macroscopic characteristics of hairs and bones following Mattioli et al. [28]–[29]. Boar remains were divided into three age-weight classes: newborn piglet (<10 kg), piglet (10–35 kg), and adult (>35 kg). Roe deer remains were classified into two classes: fawn (<1 year) and adult (>1 year). The ability of researchers to discriminate among samples from different species and age-classes was verified by means of a blind test using artificial “scat samples” containing prey remains from a variety of species and age-classes. A total of 200 samples were stored in plastic bags, each consisting of remains from one potential prey item. All potential prey in the area were represented in these samples, including hair samples from animals during both summer and winter. Each researcher was assigned 50 of these bags, chosen at random, and was assessed on their ability to correctly identify the age-class and species represented by the sample. Ability to discriminate among wild boar weight classes was additionally assessed using a further 25 samples per researcher. Only researchers who correctly identified all test samples went on to analyse true scat samples.

Most scats were entirely composed of just one prey item; the relative volume of these scats amounted to 100% of the same prey type. When more than one prey type was evident in a single scat, the relative volume of each was estimated as approximately 25, 50 or 75% of the scat's total volume. When the age class of ungulate remains could not be identified, the relative volume of the unidentified material was redistributed according to the proportions of the age-groups observed among other scats collected during the relevant period. The biomass of prey consumed to produce the collected scats was estimated using Weaver's [32] biomass model. In this model the live weight (*w_i_*) of an individual of prey type *i* is converted into *c*, an estimate of the biomass (kg) of that prey type that must have been consumed to produce one scat, according to the following equation:
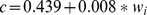
Multiplying *c* by the summed relative volumes of scats attributable to each prey species gave the inferred total biomass of each prey species consumed (hereafter, the ‘biomass consumed’), as indicated by the sample of scats collected. The weights of different age classes (obtained from data on hunted individuals in each age class) were accounted for in this calculation. The general composition of wolf diet each year was described as the percentage of total biomass consumed attributable to each prey group. These calculations were completed for the entire set of scat samples collected each year.

### Wolf dietary response and prey selection within the main, two-ungulate community

Wild boar and roe deer dominated the prey community in Alpe di Catenaia and were the main prey items of importance. To estimate selection by wolves, we focused on boar but, obviously, the complement of our estimated parameters applies to roe deer. Based on the scat analysis, we inferred the biomass consumed of boar (*C_B_*) and roe deer (*C_R_*), calculating the relative use of boar as *U_B_* = *C_B_*/(*C_B_*+*C_R_*). *U_B_* was calculated for each of the nine years and is hereafter referred to simply as boar use. The relative availability of wild boar for eight years of the study (the 2006–07 census was excluded, see above) was given by *A_B_* = *B_B_*/(*B_B_*+*B_R_*), where *B_B_* and *B_R_* are, respectively, the biomass densities of boar and roe deer in the area.

We used linear regression to model relative boar use as a function of boar availability. Consistency with the assumptions of linear regression was checked using diagnostic plots. Several studies have found seasonal differences in the absolute consumption of wild boar (percent of diet) by wolves [18], [23], [25], [41], so we initially developed models that included a seasonal component. However, season was not significant in these models so was not considered further (Text S2, Table S2).

Wolf selection for wild boar (within the wild boar-roe deer community) was assessed using Manly's standardized selection ratio, α [42]–[43]:
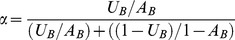
Here, α is the probability that wild boar would be selected when offered in equal biomass to roe deer. An estimate of α_i_≈0.5 indicates use of boar in proportion to boar availability. α_i_>0.5 indicates selection for wild boar, while α_i_<0.5 indicates selection against boar. We calculated Manly's selectivity index for boar for all eight years with availability estimates.

### Uncertainty estimation

Uncertainty in our estimates of wild boar use, availability, and selection by wolves within years was determined by bootstrapping [44]. For estimating boar use, all scat samples for a year were randomly sampled with replacement to produce a new estimate of the biomass consumed of both wild boar and roe deer. Similarly, for estimating boar availability, densities based on drives in separate areas of the study site were randomly sampled with replacement to produce a new estimate of density for both ungulate species. As drives in some areas each year failed to find any individuals of a given species (resulting in a density of 0 for that drive) the possibility existed for bootstrap estimates of site densities to be zero (causing analytical problems when dividing use by availability); we controlled for this by assuming a minimum possible density equal to the total number of individuals observed divided by the total area sampled that year in all drives. We used this approach to generate 4,000 bootstrap samples within each year. The relative use and relative availability of wild boar and Manly's selectivity ratio were calculated for each bootstrap sample, using the 2.5% and 97.5% quantiles to construct 95% confidence intervals around these estimates for each year. All analyses presented here were performed in R 2.13.0 [45].

## Results

### Ungulate community composition

**Figure 2:**
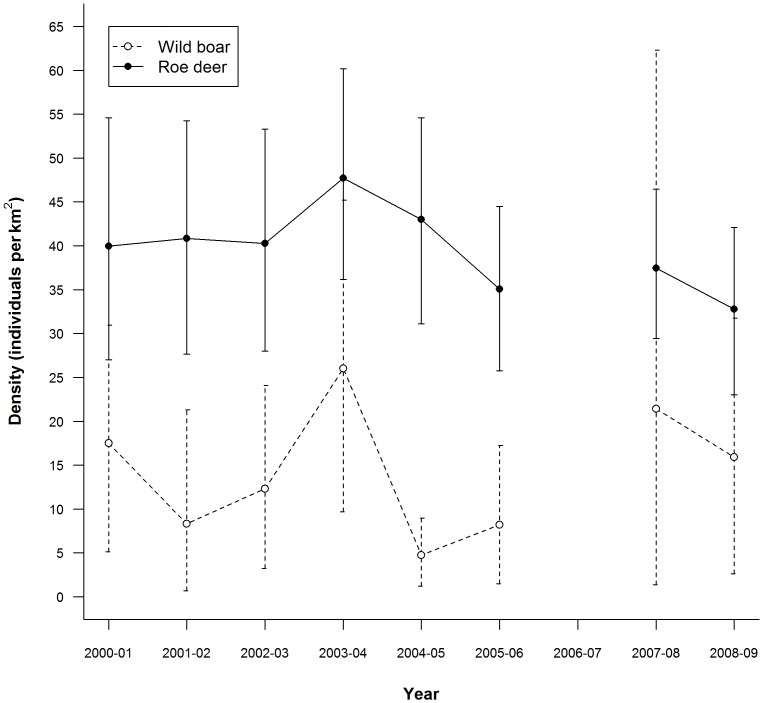
Wild boar and roe deer density in Alpe di Catenaia. The densities of the two main wolf prey items, wild boar (open circles) and roe deer (solid circles), from drive counts conducted each April in Alpe di Catenaia. Error bars represent 95% confidence intervals. Density estimates for the year 2006–07 were unavailable.

**Figure 3:**
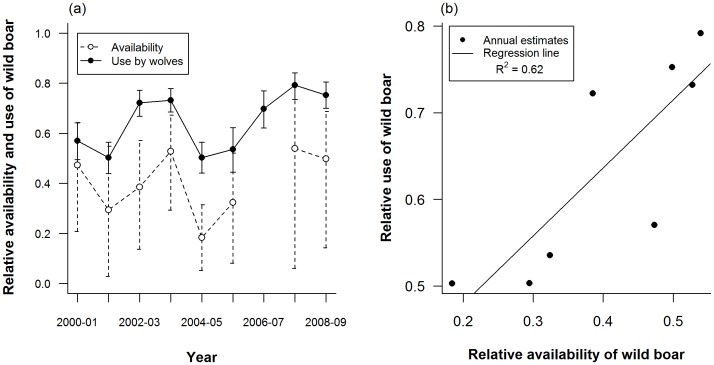
Wild boar use and availability. The relationship between the availability and use of boar (relative to ungulate community including wild boar and roe deer only) is shown. a) Relative availability (grey line, open circles) and relative use (black line, solid circles) was estimated each year from 2000–01 to 2008–09 excluding the year 2006–07. Error bars represent 95% confidence intervals. b) Linear regression analysis was used to illustrate the relationship between the relative availability and the relative use of wild boar across the eight years (solid circles) for which availability was estimated (black line, y = 0.323+0.784x, R^2^ = 0.621, *P* = 0.0124).

Wild boar density estimates ranged from 4.7 to 26 km^−2^ during the nine year study period (mean = 14.3±2.57). Roe deer density was less variable than boar density and ranged from 32.8 to 47.7 deer km^−2^ (mean = 39.6±1.64; Fig. 2). Confidence intervals, representing the uncertainty surrounding yearly density estimates due to potential sampling error, were wide for both species and made it difficult to say with confidence that densities differed among years. In fact, only the low boar density observed in 2004–05 was significantly different from other years, with 95% confidence limits that excluded the mean density observed across years. Bootstrapping simulations resulted in an exceptionally wide confidence interval for the boar density estimate for 2007–08 (Fig. 2), which reflects the high variation observed among different drives in that year (boar densities ranged from 0 to 304 km^−2^ across the 15 areas surveyed). Due to the combined uncertainty surrounding density estimates of both species, the confidence intervals surrounding our estimates of the relative availability of wild boar (based on biomass density) within this two species community were also wide and overlapped among years (Fig. 3a).

### Wolf diet and relative use of wild boar

A total of 1,974 wolf scats were collected and analyzed during the study. The diet of wolves in Alpe di Catenaia was consistently dominated by the consumption of wild boar and roe deer, which together made up 95.2±1.29% of the annual diet (Table 1). Wild boar was the primary prey, being found in the majority of scats collected, and accounting for 61.5±3.90% of biomass eaten. Roe deer, the second most prevalent prey species, accounted for 33.7±3.61% of total prey biomass. Other prey, including livestock, represented only a very small proportion of the diet (Table 1).

**Table 1:** Composition of wolf diet was assessed based on scat samples collected in Alpe di Catenaia, Italy.

		Wolf diet composition from 2000 through 2009: percentage of biomass consumed per prey item									
		2000–01^b^	2001–02	2002–03	2003–04	2004–05	2005–06	2006–07	2007–08	2008–09	
Prey item	Scat samples^a^	178	242	262	293	232	143	144	208	272	Mean ± SE (n = 9)
Wild boar	1284	55.9	48.2	68.5	71.2	48.8	46.1	68.7	76.5	69.6	61.5±3.90
Roe deer	804	42.1	47.6	26.3	26.1	48.2	39.9	29.8	20.1	22.9	33.7±3.61
Red deer	12	0	0	0	0	0	0	0	0.4	6.1	0.7±0.67
Hare	26	0	0	0.6	1.8	1.1	4.5	0.6	1.0	0	1.1±0.47
Small rodents	18	0.4	0.2	0.3	0.4	1.2	0	0	0	0.3	0.3±0.12
Sheep	29	1.6	3.7	4.3	0.5	0.8	8.5	0	0	0.3	2.2±0.95
Goat	3	0	0.4	0	0	0	1.0	1.0	0	0	0.3±0.14
Cattle	3	0	0	0	0	0	0	0	2.0	0.8	0.3±0.22

a Scat samples per year sum to the total number of samples used in all analysis over 9 years (1,974). Scat samples per prey item are defined as the total number of scats found containing that prey item in any proportion and may, therefore, sum to more than the total number of scat samples collected.

b For analysis purposes our data years began in May and ended in April; the 2000–01 year represents all scats collected between 1 May 2000 and 30 April 2001.

Although boar and roe deer consistently accounted for over 90% of biomass eaten, the percent of diet individually attributable to either species was variable across the nine year study period (Table 1); this is reflected in our estimates of boar use by wolves (Fig. 3a). Boar use (mean over the entire period: 0.65±0.039; Fig. 3a) was generally higher than that of roe deer and, for five of the years analyzed, the percent of wolf diet made up of wild boar was more than twice that of roe deer. Confidence intervals surrounding estimates of boar use were narrow in comparison to those calculated for boar availability (Fig. 3a), reflecting the large number of scats collected each year during the study (>140 scats each year compared to only 9–15 drives per year that were used to estimate availability).

Inter-annual fluctuations in boar use, the proportional biomass of wild boar in wolf diet relative to that of roe deer and wild boar combined, reflected changes in the proportional availability of wild boar as a prey item. Based on the regression of boar use as a function of availability, boar availability accounted for 62% of the variation in boar use across years (β_BA_ = 0.784±0.2222, R^2^ = 0.621, *t*_6_ = 3.529, *P* = 0.012; Fig. 3b). The years of comparably low boar use (2001–02, 2004–05, and 2005–06; Fig. 3a) coincided with years of low boar density, rather than years of high roe deer density (Table 1, Fig. 2).

### Prey Selection

**Figure 4:**
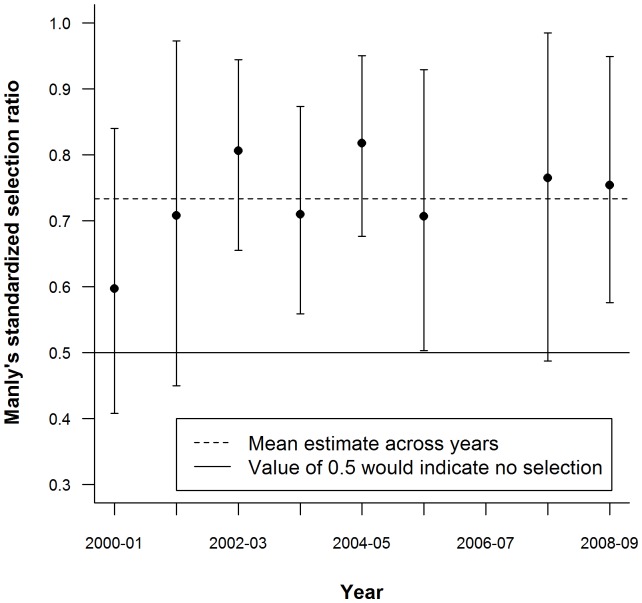
Uncertainty and variation in the selection of wild boar across years. Manly's standardized selection ratio for wild boar (in wolf diet) was calculated for eight years from 2000–01 to 2008–09. This index is based on the relative availability and use of boar within the main two-prey community composed only of wild boar and roe deer. Error bars representing bootstrapped 95% confidence intervals are displayed. Values approximately equal to 0.5 (black line) indicate prey use in proportion to availability in a two-prey system while selection for and against wild boar are indicated by higher and lower values respectively. The mean value of Manly's selection ratio for boar during the study period was 0.733±0.0234 (dashed line).

Estimates of Manly's selectivity index ranged between 0.60 and 0.82 across eight years with a mean of 0.73±0.023 indicating a strong tendency for selection for boar and against roe deer by the wolves in Alpe di Catenaia (Table 2). Estimates of Manly's index indicated selection for boar (α_Boar_>0.5) in five out of the eight years examined (Table 2). This reflects the fact that boar use was generally high relative to its availability (Fig. 3a). The confidence intervals for the yearly estimates of Manly's index were wide, representing a high level of uncertainty due to sampling variation among individual scats and drive censuses. The overlap of confidence intervals among years cautions against the temptation to infer variation in selection for boar during the study period (Fig. 4).

**Table 2:** Selection of wild boar as a prey species based on estimates of boar use by wolves and relative availability within Alpe di Catenaia, Italy.

Year^a^	Scat samples collected	Relative wild boar availability^b^	Relative wild boar use	Manly's standardized selection ratio, calculated for wild boar use in wolf diet^c^	Bootstrapped 95% confidence intervals on Manly's standardized selection ratio	
					Lower limit	Upper limit
2000–01	178	0.47	0.57	0.60	0.41	0.84
2001–02	242	0.30	0.50	0.71	0.45	0.97
2002–03	262	0.39	0.72	0.81	0.66	0.94
2003–04	293	0.53	0.73	0.71	0.56	0.87
2004–05	232	0.18	0.50	0.82	0.68	0.95
2005–06	143	0.32	0.54	0.71	0.50	0.93
2006–07	144	Not available	0.70	NA	NA	NA
2007–08	208	0.54	0.79	0.77	0.49	0.99
2008–09	272	0.50	0.75	0.75	0.58	0.95
Mean ± SE		0.40±0.043	0.65±0.039	0.73±0.023		

a Data years began in May and ended in April; the 2000–01 year represents all scats collected between 1 May 2000 and 30 April 2001.

b Wild boar availability and use in wolf diet are calculated based on biomass (kg per km^2^) relative to the availability and use of the main ungulate community in Alpe di Catenaia consisting of wild boar and roe deer only. See methods for more detail.

c For Manly's standardized selection ratio, values approximately equal to 0.5 indicate prey use in proportion to availability in a two-prey system while selection for and against the prey type of focus would be indicated by higher and lower values respectively.

## Discussion

We found that the consumption of wild boar dominated wolf diet and the use of boar as prey (relative to the use of roe deer) is strongly related to the relative availability of wild boar within the study area. Wolves in Alpe di Catenaia selected wild boar over roe deer as prey and there is little evidence of variation in the strength of this selection among years. Had we not recognized the uncertainty inherent in our data we may have erroneously interpreted variation in our estimates of prey selection as indicative of differential selection among years. The length of our study combined with our large sample size of scats (1,974 over the nine year study period) allowed us not only to examine inter-annual variation in wolf predation, but also to consider the potential impacts of sampling error on our results. The amalgamation of uncertainty from multiple sources (i.e. the estimation of both prey availability and use) means that the uncertainty surrounding final estimates of prey selection is very large. Accounting for this uncertainty limited the conclusions we were able to make but ensured that our interpretation of inter-annual variability in prey selection by wolves in Alpe di Catenaia was fully supported by the data.

### Wolf diet in Alpe di Catenaia

As in other areas with an abundance of wild prey [11], [18], [22], [28], [46], the wolves inhabiting Alpe di Catenaia site subsist mainly on wild ungulates, with a very low frequency of livestock predation. It is the selection of prey species within the wild ungulate community that appears somewhat unusual. In contrast to wolves in other parts of Europe which often avoid boar as prey [11], wolves in Alpe di Catenaia appear to rely heavily on wild boar. Despite the wide confidence intervals surrounding our annual estimates of boar selection we found that boar were selected (over roe deer) in six of the eight years examined. Boar made up the majority of biomass eaten throughout most of the study period. While we cannot be certain of a causal relationship, the strength of boar availability as a predictor of boar use suggests that wolf diet was tracking the fluctuations in boar densities. Roe deer, while an important prey item, usually made up a smaller portion of wolf diet. The percentage biomass of roe deer in wolf diet appeared to peak when boar densities were low, not when roe deer densities were highest. In Alpe di Catenaia, the relatively stable roe deer population may represent an alternative prey source which suffers higher predation when wild boar densities decline. That the extent of wolf predation on roe deer can fluctuate widely, even when roe deer are relatively stable, underlines the importance of taking a community perspective to investigate and predict predation impacts on any given species [10], [47].

The strength of selection for boar in Alpe di Catenaia raises the question of why similar selectivity is not seen throughout Europe. There could be three reasons for this. Firstly, many European ungulate communities include red deer, which appear to be a favoured prey of wolves in many sites (reviewed by Okarma [11]). The scarcity of red deer (completely absent until 2007) in Alpe di Catenaia could lead to stronger selection for wild boar and could drive the dietary response of wolves to changes in boar availability observed in this study. A study of wolves in another region of the same Italian Province but with a more diverse prey community (including red and fallow deer in addition to roe deer and wild boar [29]), found that while wolves relied heavily on boar consumption, the composition of wolf diet was unrelated to boar availability. Secondly, wild boar in Mediterranean areas are relatively small; for example, adult boar in Alpe di Catenaia, weighed 66.5±0.48 kg (based on mass data for 1,286 adult boar carcasses collated by the Province of Arezzo). In central Europe, where adult male boar can exceed 300 kg in size [48], their active defence behaviour can, reportedly, make them dangerous prey for wolves [49]. This small size of adults in Alpe di Catenaia may make boar less threatening as prey and, in combination with their large litter sizes (often exceeding 5 piglets per litter [50]) and grouping behaviour, may encourage wolves to select boar over roe deer [22], [28]–[29]. Finally, this study included only a small number of wolves, believed to belong to a single pack, and therefore it is possible that the preference for boar reflects the habits of this particular pack or the individuals within it. However, similar studies in the region have also identified a preference of wolves for wild boar over roe deer [29], [51]. Individual preferences could lead to variation in selection for prey among years but we found no evidence of significant interannual variation in this study (see below for further discussion).

### The importance of intra-annual uncertainty when considering variation in prey selection

Variation in wolf predation patterns (e.g. disparate prey selection among sites with similar prey communities) may reflect underlying differences in the ecology of distinct sites or a failure to assess accurately the uncertainty inherent in estimates of wolf feeding habits. Our final estimates of prey selection indices had very wide confidence intervals, suggesting high levels of uncertainty in the data on boar use (from wolf scats) and, in particular, the data on boar availability (from drive censuses). Sampling error is difficult to avoid and is present in all datasets, to some extent. Uncertainty in this study arose particularly from the estimation of annual prey densities, because of the low number of ‘density samples’ (drive censuses from different areas of the study site) in each year. This is a common situation in European ungulate research and many datasets will incorporate similar levels of uncertainty in their density estimates.

Without considering uncertainty, our results would suggest substantial variation among years in the strength of selection for boar by the wolves in Alpe di Catenaia. However, when we put the observed variation into the context of within-year uncertainty it is not possible to say with any confidence that prey selection in our site differed from one year to the next. This finding also compels caution when comparing selectivity estimates between different sites. For example, comparing the point estimates of Manly's α from this study to those observed in other areas could suggest geographic variation in selection (especially if the studies being compared were of short duration or if results had been pooled across years). We do not suggest that such variation does not exist but, in some cases, reported differences in wolf predatory habits among sites (or time periods within sites) might disappear when uncertainty in estimated metrics (such as selection indices) is accounted for.

### Caveats and considerations for future research

Our findings should be considered in light of several important caveats. The first two relate to the fact that only one census of prey was possible each year. While the prey selection observed in this study could arise for the reasons described above (relating to community composition and boar body size) it could also be partially driven by variation in prey vulnerability due to temporal fluctuations in population age structure. In particular, because wild boar can produce two litters within a single year and boar piglets are likely to be more vulnerable as prey, there is a high potential for both inter- and intra-annual variation in the overall vulnerability of wild boar [52]. Estimating the age structure of prey populations multiple times each year would help isolate the influence of changing prey vulnerability on selection by wolves. Additionally, seasonal movement of prey species could affect their relative availability, and such intra-annual variation will not be reflected by annual drive censuses. However, telemetry studies at the site suggest that the mean home range areas (Minimum Convex Polygons) of the prey species (roe deer: 4.0±4.43 km^2^, n = 69 individuals; wild boar: 7.5±9.50 km^2^) were substantially smaller than the study site (120 km^2^), suggesting that such intra-annual migration was unlikely to be a major factor.

Three further caveats suggest general lessons for studies of dietary selectivity.

Firstly, we do not know how much of the prey consumption we observed could be due to scavenging upon carcasses rather than direct predation. In the future, closer observation of individual wolves, using radio-telemetry, may provide estimates of scavenging frequency and allow us to adjust our estimates of predation accordingly. Secondly, all density estimation methods incorporate some degree of error due to unobserved individuals and the drive censuses used in this study are no exception. McCullough [53] estimated that errors in drive census estimates can be as large as 20–30% of the true population size. Estimates of wild boar densities are particularly challenging due to their wide-ranging behavior and aggregated distributions [54]. Capture-mark-recapture estimates might provide more accuracy but can be more resource intensive (in terms of time, equipment and labor). When mark-capture-recapture estimates are not possible, researchers can form more robust conclusions from studies requiring density estimates by acknowledging the uncertainties associated with chosen methods and, when possible, by comparing estimates based on a variety of methods (e.g. pellet counts, camera surveys etc.) simultaneously. Finally, on a related note, our spring density estimates took place before the birth of new roe deer fawns but after the initial pulse of boar births. This means that we might be over-estimating the relative availability of boar within this two-prey system and therefore under-estimating the strength of selection for boar as prey. Our conservative estimates of boar selection would most likely be strengthened if we were able to use post-reproductive roe deer densities. In the future, this bias could be avoided by either using estimates of roe deer reproduction to estimate post-reproductive densities or by surveying ungulate densities later in the spring.

### Conclusion

Wild boar are the primary prey of wolves in Alpe di Catenaia, Italy. For the wolves in this area, roe deer represent an alternative prey source which increases in dietary importance when boar densities decline. While accounting for sampling uncertainty in our data, we were able to show that boar were significantly selected for during the majority of the years studied. Boar use throughout the study period was strongly related to the relative availability of wild boar within this predominantly two-prey community, a finding which suggests a dietary response by wolves to the availability of wild boar. The high natural variability of wild boar populations [52], [55] thus could have important ramifications for predator impacts on roe deer.

Our findings demonstrate that failing to account for uncertainty when interpreting inter-annual variation in studies of predator diet might lead to conclusions that are not fully supported by the data. In addition to presenting multi-year datasets without pooling data across years, when possible, future studies of prey selection should strive to account for possible sources of uncertainty due to sampling procedures. While the comparison of a predator's dietary composition and prey selection across years and sites can yield important information about large-scale patterns of predation, such analyses often incorporate uncertainty from multiple sources. Caution must be taken to describe such uncertainty before drawing ecological conclusions, so that the nature of complex predator-prey relationships is properly represented.

## Supporting Information

Text S1 **Literature reviewed on European wolf diet (see Table S1).**
(DOC)

Text S2 **The analysis of boar use in response to season (methods and results).**
(DOC)

Table S1 **Published studies of wolf diet in Europe surveyed for analysis of uncertainty and inter-annual variability in estimates of dietary composition and prey selection.**
(DOC)

Table S2 **Repeated measure ANOVA of the effects of boar availability, season and their interaction on seasonal use of boar by wolves (relative to the wild ungulate community including boar and roe deer).** Data were collected from 2000–2009 in the Alpe di Catenaia study site in Italy.
(DOC)

Contract S1 **Contract for ungulate work, 2005.**
(PDF)

Contract S2 **Contract for ungulate work, 2009.**
(PDF)

Contract S3 **Contract for wolf work, 2000.**
(PDF)

Contract S4 **Contract 1 for wolf work, 2001–03.**
(PDF)

Contract S5 **Contract 2 for wolf work, 2001–03.**
(PDF)

Contract S6 **Contract for wolf work, 2004.**
(PDF)

Contract S7 **Contract for wolf work, 2005–07.**
(PDF)

Contract S8 **Contract for wolf work, 2008–09.**
(PDF)

Contract S9 **Contract for wolf work, 2009–2013.**
(PDF)
