# Supplementary material for: Prey Selection by an Apex Predator: The Importance of Sampling Uncertainty
Source: PLoS One. 2012 Oct 26;7(10):e47894. doi: 10.1371/journal.pone.0047894 (PMC3482236; doi:10.1371/journal.pone.0047894)
Supplement: Text S1 — Literature reviewed on European wolf diet (see Table S1). (DOC) [file pone.0047894.s001.doc]

**Text S1: Literature reviewed on European wolf diet (see Table S1).**

1. Ansorge H, Kluth G, Hahne S (2006) Feeding ecology of wolves *Canis lupus* returning to Germany. Acta Theriologica 51: 99-106.

2. Barja I (2009) Prey and prey-age preference by the Iberian wolf *Canis lupus* signatus in a multiple-prey ecosystem. Wildlife Biology 15: 147-154.

3. Capitani C, Bertelli I, Varuzza P, Scandura M, Apollonio M (2004) A comparative analysis of wolf (*Canis lupus*) diet in three different Italian ecosystems. Mammalian Biology 69: 1-10.

4. Ciucci P, Boitanti L, Pelliccioni ER, Rocco M, Guy I (1996) A comparison of scat-analysis methods to assess the diet of the wolf *Canis lupus*. Wildlife Biology 2: 37-48.

5. Cuesta L, Barcena F, Palacios F, Reig S (1991) The trophic ecology of the Iberian wolf (*Canis-lupus*-signatus, Cabrera, 1907) - a new analysis of stomachs data. Mammalia 55: 239-254.

6. Gazzola A, Bertelli I, Avanzinelli E, Tolosano A, Bertotto P, et al. (2005) Predation by wolves (*Canis lupus*) on wild and domestic ungulates of the western Alps, Italy. Journal of Zoology 266: 205-213.

7. Jedrzejewski W, Jedrzejewska B, Okarma H, Schmidt K, Zub K, et al. (2000) Prey selection and predation by wolves in Bialowieza Primeval Forest, Poland. Journal of Mammalogy 81: 197-212.

8. Jedrzejewski W, Jedrzejewska B, Okarma H, Ruprecht AL (1992) Wolf predation and snow cover as mortality factors in the ungulate community of the Bialowieza-Natural-Park, Poland. Oecologia 90: 27-36.

9. Lesniewicz K, Perzanowski K (1989) The winter diet of wolves in Bieszczady Mountains. Acta Theriologica 34: 373-380.

10. Macdonald DW, Boitani L, Barrasso P (1980) Foxes *Vulpes-vulpes* wolves *Canis-Lupus* and conservationin the Abruzzo Mountains Italy. Zimen, E. pp. P223-236.

11. Marucco F, Pletscher DH, Boitani L (2008) Accuracy of scat sampling for carnivore diet analysis: Wolves in the Alps as a case study. Journal of Mammalogy 89: 665-673.

12. Mattioli L, Apollonio M, Mazzarone V, Centofanti E (1995) Wolf food habits and wild ungulate availability in the Foreste Casentinesi National Park, Italy. Acta Theriologica 40: 387-402.

13. Mattioli L, Capitani C, Gazzola A, Scandura M, Apollonio M (2011) Prey selection and dietary response by wolves in a high-density multi-species ungulate community. European Journal of Wildlife Research 57: 909-922.

14. Meriggi A, Rosa P, Brangi A, Matteucci C (1991) Habitat use and diet of the wolf in Northern Italy. Acta Theriologica 36: 141-151.

15. Meriggi A, Brangi A, Matteucci C, Sacchi O (1996) The feeding habits of wolves in relation to large prey availability in northern Italy. Ecography 19: 287-295.

16. Nores C, Llaneza L, Alvarez MA (2008) Wild boar *Sus scrofa* mortality by hunting and wolf *Canis lupus* predation: an example in northern Spain. Wildlife Biology 14: 44-51.

17. Olsson O, Wirtberg J, Andersson M, Wirtberg I (1997) Wolf *Canis lupus* predation on moose Alces alces and roe deer Capreolus capreolus in south-central Scandinavia. Wildlife Biology 3: 13-25.

18. Patalano M, Lovari S (1993) Food-habits and trophic niche overlap of the wolf *Canis-lupus*, L 1758 and the red fox *Vulpes-vulpes* (L 1758) in a Mediterranean mountain area. Revue D Ecologie-La Terre Et La Vie 48: 279-294.

19. Pezzo F, Parigi L, Fico R (2003) Food habits of wolves in central Italy based on stomach and intestine analyses. Acta Theriologica 48: 265-270.

20. Reig S, de la Cuesta L, Palacios F (1985) The impact of human activities on the food habits of red fox and wolf in Old Castille, Spain. Terre Et La Vie 40: 151-155.

21. Salvador A, Abad PL (1987) Food-habits of a wolf population (*Canis-lupus*) in Leon Province, Spain. Mammalia 51: 45-52.

22. Smietana W, Klimek A (1993) Diet of wolves in the Bieszczady Mountains, Poland. Acta Theriologica 38: 245-251.

23. Valdmann H, Andersone-Lilley Z, Koppa O, Ozolins J, Bagrade G (2005) Winter diets of wolf *Canis lupus* and lynx *Lynx lynx* in Estonia and Latvia. Acta Theriologica 50: 521-527.

24. Zunna A, Ozolins J, Pupila A (2009) Food habits of the wolf *Canis lupus* in Latvia based on stomach analyses. Estonian Journal of Ecology 58: 141-152.
