# Supplementary material for: Prey Selection by an Apex Predator: The Importance of Sampling Uncertainty
Source: PLoS One. 2012 Oct 26;7(10):e47894. doi: 10.1371/journal.pone.0047894 (PMC3482236; doi:10.1371/journal.pone.0047894)
Supplement: Text S2 — The analysis of boar use in response to season (methods and results). (DOC) [file pone.0047894.s002.doc]

**Text S2: The analysis of boar use in response to season (methods and results)**

When collecting scats we also recorded the ‘season’ of collection. While collected year-round (see Methods) we classified scats into two seasons: ‘summer’ lasting from May to October and ‘winter’ from November to April. Over the 9 year study period we collected 802 “summer” scats and 1172 “winter” scats. When near the limits of these seasonal periods special care was taken to assess the age of the scat sample and likely date of deposition. The decision to divide the year into these two periods was made *a priori* because of the biology of both predator and prey species. During the “summer” season wolves have new pups and pack activities are therefore localized around den areas (contrasting the winter when wolves are more nomadic). Also during the summer period, both wild boar and roe deer are more likely to have young which make vulnerable prey. All these factors could contribute to distinct patterns of selection between these two seasons.

We estimated the seasonal biomass consumed of boar and roe deer based on the scats from each season throughout the nine year study period. We then calculated the relative use of boar (within the two-prey community including boar and roe deer), *U_B_*, separately for each summer and winter. All calculations followed the procedure described in the main text for assessment of wolf diet and prey use (see Methods). We modeled seasonal values of boar use (16 estimates of use across eight years, based on summer and winter scat samples) as a response to season, annual availability (*U_A_,* see main text for methods) and their interaction. We tested the significance of these parameters using a repeated measure ANOVA with year as the error term; this allowed us to control for only having one measure of availability per year. We found the effects of season on relative boar use to be non-significant (Table S2). We did not consider seasonal variation further in our analyses, therefore, but modeled the annual estimates of boar use (based on all scats collected each year) as a response to the relative availability of this species.
