# Supplementary material for: Prey Selection by an Apex Predator: The Importance of Sampling Uncertainty
Source: PLoS One. 2012 Oct 26;7(10):e47894. doi: 10.1371/journal.pone.0047894 (PMC3482236; doi:10.1371/journal.pone.0047894)
Supplement: Table S1 — Published studies of wolf diet in Europe surveyed for analysis of uncertainty and inter-annual variability in estimates of dietary composition and prey selection. (DOC) [file pone.0047894.s003.doc]

**Table S1: Published studies of wolf diet in Europe surveyed for analysis of uncertainty and inter-annual variability in estimates of dietary composition and prey selection.**

| Source | Study details | | |  | Dietary composition | |  | Prey selection | | |
| --- | --- | --- | --- | --- | --- | --- | --- | --- | --- | --- |
|  | Country | Method ^a^ | Length (years) |  | Calculated  intra-annual uncertainty | Examined inter-annual variability |  | Evaluated in study | Calculated intra-annual Uncertainty | Examined inter-annual variability |
| Ansorge et al. [1] | Germany | S | 3 |  | No | No |  | Yes | No | No |
| Barja [2] | Spain | S | 5 |  | No | Yes |  | Yes | No | No |
| Capitani et al. [3] | Italy | S | 1 |  | No | NA |  | Yes | No | NA |
| Ciucci et al. [4] | Italy | S | 2 |  | No | No |  | No | NA | NA |
| Cuesta et al. [5] | Spain | OS | 15 |  | No | No |  | No | NA | NA |
| Gazzola et al. [6] | Italy | S | 3 |  | No | No |  | Yes | No | No |
| Jedrzejewski et al. [7-8] | Poland | KS | 11 |  | No | Yes |  | Yes | No | No |
| Lesniewicz and Perzanowski [9] | Poland | O | 2 |  | No | No |  | No | NA | NA |
| Macdonald [10] | Italy | S | 3 |  | No | No |  | No | NA | NA |
| Marucco et al. [11] | Italy | KS | 3 |  | Yes | Yes |  | Yes | Yes | Yes |
| Mattioli et al. [12-13] | Italy | S | 11 |  | No | Yes |  | Yes | Yes | Yes |
| Meriggi et al. [14] | Italy | S | 1 |  | No | No |  | No | NA | NA |
| Meriggi et al. [15] | Italy | S | 5 |  | No | Yes |  | Yes | No | No |
| Nores et al. [16] | Spain | S | 1 |  | No | NA |  | No | NA | NA |
| Olsson et al. [17] | Sweden & Norway | S | 4 |  | No | No |  | No | NA | NA |
| Patalano and Lovari [18] | Italy | S | 2 |  | No | Yes |  | No | NA | NA |
| Pezzo et al. [19] | Italy | O | 5 |  | No | No |  | No | NA | NA |
| Reig et al. [20] | Spain | O | 4 |  | No | No |  | No | NA | NA |
| Salvador and Abad [21] | Spain | S | 2 |  | No | Yes |  | No | NA | NA |
| Smietana and Klimek [22] | Poland | S | 3 |  | No | No |  | No | NA | NA |
| Valdmann et al. [23] | Latvia & Estonia | OS | 4 |  | No | No |  | No | NA | NA |
| Zunna [24] | Latvia | O | 6 |  | No | No |  | No | NA | NA |

^a^ The methods of dietary analysis used in each study were broadly categorized. Studies generally relied on samples of scats (S), internal organs (O) (i.e. stomachs and intestines from killed wolves), or carcasses found of prey killed by wolves (K). Some studies combined more than one method.
