# Supplementary material for: Prey Selection by an Apex Predator: The Importance of Sampling Uncertainty
Source: PLoS One. 2012 Oct 26;7(10):e47894. doi: 10.1371/journal.pone.0047894 (PMC3482236; doi:10.1371/journal.pone.0047894)
Supplement: Table S2 — Repeated measure ANOVA of the effects of boar availability, season and their interaction on seasonal use of boar by wolves (relative to the wild ungulate community including boar and roe deer). Data were collected from 2000–2009 in the Alpe di Catenaia study site in Italy. (DOC) [file pone.0047894.s004.doc]

Table S2: Repeated measure ANOVA ^a^ of the effects of boar availability, season and their interaction on seasonal use of boar by wolves (relative to the wild ungulate community including boar and roe deer) ^b^. Data were collected from 2000-2009 in the Alpe di Catenaia study site in Italy.

| Model parameters ^ab^ | Num. Df | Denom. Df | Sum of Sq. | MS | Res. MS | F | *P* (> F) |
| --- | --- | --- | --- | --- | --- | --- | --- |
| Boar availability | 1 | 6 | 0.145 | 0.145 | 0.007 | 20.595 | 0.004 |
| Season | 1 | 6 | 0.006 | 0.006 | 0.006 | 1.020 | 0.352 |
| Boar availability * Season | 1 | 6 | 0.006 | 0.006 | 0.006 | 1.095 | 0.336 |

^a^ Year was included as the error term when testing for effect of boar availability as there was only one measure of availability per year.

^b^ Wild boar availability and use in wolf diet are calculated based on biomass (kg per km^2^) relative to the availability and use of the main ungulate community in Alpe di Catenaia consisting of wild boar and roe deer only. Boar use was calculated seasonally based on collected wolf scats. Seasons were defined as either summer (May to October) or winter (November to April). Boar availability was calculated using annual estimates of ungulate density based on drive censuses. See methods for more detail.
