# Supplementary material for: Prey Selection by an Apex Predator: The Importance of Sampling Uncertainty
Source: PLoS One. 2012 Oct 26;7(10):e47894. doi: 10.1371/journal.pone.0047894 (PMC3482236; doi:10.1371/journal.pone.0047894)
Supplement: Contract S1 — Contract for ungulate work, 2005. (PDF) [file pone.0047894.s005.pdf]

REGIONE TOSCANA-GIUNTA REGIONALE

DIREZIONE GENERALE SVILUPPO ECONOMICO

AREA DI COORDINAMENTO POLITICHE PER LO SVILUPPO RURALE

SETTORE TUTELA DELLA FAUNA E GESTIONE FAUNISTICO VENATORIA, MECCANIZZAZIONE AGRICOLA, CALAMITA' NATURALI, SISTEMI IRRIGUI

Dirigente Responsabile: Paolo Banti

**Decreto**	**N° 7182**	del **21 Dicembre 2005**

Pubblicità/Pubblicazione: Atto soggetto a pubblicazione per estratto

Allegati n°: 0

Oggetto:
L.R.3/94 Concessione alla Provincia di Arezzo di un contributo speciale finalizzato alla realizzazione di un progetto relativo alla diffusione del Cinghiale , il suo rapporto con il predatore lupo, i danni apportati alla fauna, al bosco e all'agricoltura. Impegno della spesa

MOVIMENTI CONTABILI

Capitolo	*Anno*	*Tipo Movimento.*	*Numero*	*Var.*	*Data*	*Importo in Euro*
U-27051	2005	Impegno/Assegnazione	6444		23-12-2005	30.400,00

Atto soggetto al controllo interno ai sensi della D.G.R. n. 1315/2003

Controllo eseguito senza rilievi.
Atto certificato il 02-01-2006

IL DIRIGENTE

Vista la legge 11 febbraio 1992, n. 157 recante "Norme per la protezione della fauna selvatica omeoterma e per il prelievo venatorio;

Vista la legge regionale 12 gennaio 1994, n. 3 di recepimento della 157/92;

Visto l'art.3 della L.R 17 marzo 2000, n.26 e successive modifiche ed integrazioni;

Vista la L.R. 5 Agosto 2003, n. 44 ed in particolare l'art. 8;

Visto il decreto n. 5740 del 27.10.2005 con il quale il sottoscritto è stato nominato responsabile del Settore "Tutela della fauna e gestione faunistico venatoria, meccanizzazione agricola, calamità naturali, sistemi irrigui";

Visto il capitolo 27051 del bilancio che prevede un fondo a disposizione della Giunta Regionale per iniziative di interesse regionale in favore dell'ambiente e della fauna, per attività di educazione e propaganda, nonché per eventuali contributi ad enti o associazioni operanti nel settore per l'espletamento dei compiti propri della Giunta Regionale;

Vista la richiesta della Provincia di Arezzo di un contributo straordinario per la realizzazione di un progetto di ricerca faunistica di interesse regionale sul problema della diffusione del cinghiale, il suo rapporto con il predatore lupo e i danni apportati alla fauna, al bosco ed all'agricoltura nella zona dell'Alpe di Catenaia ;

Valutata positivamente tale iniziativa e ritenuto che la stessa risponde all'interesse generale di tutela e conservazione della fauna e che i dati e le informazioni emerse oltre a confluire nell'aggiornamento del Piano Faunistico Venatorio provinciale , consentono una migliore gestione delle oasi di protezione faunistica ;

Richiamata la deliberazione della Giunta regionale n. 674 del 03.09.1992 di determinazione dei criteri e delle modalità di cui all'articolo 12 della Legge 241/90 per la concessione di contributi, sussidi ed ausili finanziari a persone, Enti pubblici e privati;

Considerato che nella suddetta deliberazione è previsto che per iniziative di carattere regionale può essere erogato un contributo fino ad un massimo del 80% della spesa prevista;

Ritenuto concedere un contributo complessivo di Euro 30.400,00 per la realizzazione dell'iniziativa pari all'80% dell'importo totale previsto pari a 38.000,00 euro, importo ritenuto congruo in rapporto alle attività previste;

Considerato che il contributo non è soggetto a ritenuta d'acconto ai sensi dell'articolo 26 del DPR 600/73 in quanto trattasi di Ente non a scopo di lucro;

Vista la L.R. n. 72 del 20/12/2004, con la quale è stato approvato il Bilancio regionale di previsione per l'esercizio finanziario 2005 ed il Bilancio pluriennale 2006/2007;

Vista la Deliberazione G.R. n.1385 del 27/12/2004 di approvazione del Bilancio gestionale 2005/2007;

Vista la Deliberazione G.R. n.1141 del 28/11/2005 "Variazione al Bilancio Gestionale 2005 e pluriennale 2005/2007 ai sensi degli artt. 22,23 e 24 della L.R.36/2001;

DECRETA

- di concedere alla Provincia di Arezzo, per i motivi di cui in premessa, un contributo per la realizzazione del progetto di ricerca faunistica di interesse regionale sulla popolazione del cinghiale di Euro 30.400,00 pari all'80% dell'importo complessivo stimato in Euro 38.000,00;

- di assumere sul capitolo 27051 del bilancio di previsione dell'esercizio corrente, che presenta la necessaria disponibilità, l'impegno di Euro 30.400,00 a favore della Provincia di Arezzo;

- di liquidare la somma impegnata con il presente atto, secondo le modalità previste dall'art. 44 e 45 DPGR n. 61/r del 19 dicembre 2001, dietro presentazione di regolare rendicontazione;

- di procedere all'iscrizione del suddetto contributo nell'elenco dei beneficiari ai sensi del DPR 118/2000;

Il presente provvedimento, soggetto a pubblicità ai sensi della legge regionale 9/95 in quanto atto conclusivo di procedimento amministrativo regionale, è pubblicato per estratto sul Bollettino Ufficiale della Regione Toscana ai sensi dell'articolo 3, comma 2 della legge regionale 18/96 e successive modifiche ed integrazioni.

Il Dirigente

PAOLO BANTI
