# Supplementary material for: Prey Selection by an Apex Predator: The Importance of Sampling Uncertainty
Source: PLoS One. 2012 Oct 26;7(10):e47894. doi: 10.1371/journal.pone.0047894 (PMC3482236; doi:10.1371/journal.pone.0047894)
Supplement: Contract S2 — Contract for ungulate work, 2009. (PDF) [file pone.0047894.s006.pdf]

\\ \ Prov. AR - Capriolo

PROVINCIA
DI AREZZO

Caccia e pesca

Caccia e pesca

Piazza della Libertà, 3
52100 Arezzo

Telefono +39 0575 392270
Fax +39 0575 392425
E-Mail mguffanti@provincia.arezzo.it
Sito web www.provincia.arezzo.it

C.F. 80000610511
P.IVA 00850580515

ISO 9001:2000

All'Università di Sassari
Dipartimento di Zoologia e
Genetica Evoluzionistica

c. a. Prof. Marco Apollonio

Via Muroni, 25
07100 Sassari

Prot. N. 221112 / 42.26.00.03 del 17-11-2009

Oggetto: Progetto "Influenza delle attività antropiche sui ritmi di attività, uso dello spazio e selezione dell'Habitat del capriolo in ambiente appenninico".

Caro Professore,

sono lieto di comunicarle che la Regione Toscana ha approvato con Decreto n. 5404 del 29 Ottobre 2009, il progetto di cui all'oggetto per un'importo complessivo annuo di € 25.000,00. Come a lei già noto, il progetto, cofinanziato per il 51% da questa Provincia di Arezzo, verrà svolto in collaborazione con il Centro di Ricerca per la Selvicoltura. L'importo per l'anno 2009, a disposizione dell'Ente da lei rappresentato, è di € 18.000,00 come da prospetto allegato, e dovrà essere speso unicamente per le voci e nei valori di seguito riportati:

- Spese generali € 800,00
- Personale € 15.000,00;
- Beni di consumo € 1.700,00;
- Beni inventariabili € 500,00

Il punto 7. "Tipologie di spese ammissibili", dell'Allegato A del Bando contenente le disposizioni tecniche e procedurali per l'attuazione della Misura 6.2.14 del Piano Agricolo Regionale 2009-2010 (Del. Cons. Regionale n. 98/2008), fa presente che "*Sono ammissibili esclusivamente le spese, debitamente documentate e quietanziate, direttamente riconducibili all'iniziativa. Le spese devono sempre essere comprovate da fatture o da altri documenti aventi forza probante equivalente, esclusivamente intestate al soggetto beneficiario del contributo, in cui sia sempre specificata la natura e la quantità del bene acquistato o della prestazione resa*".

Inoltre al fine di ottenere dalla Regione Toscana, un'anticipo pari al 50% del contributo assegnato, si chiede cortesemente di inviare all'Ufficio Caccia di questa Provincia, una dichiarazione, fatta in accordo con l'ente collaboratore al progetto, dalla quale risulti la data d' inizio dell'iniziativa stessa.

In attesa di un riscontro da parte sua porgo distinti saluti

Distinti saluti

Il Coordinatore
Dott. Gabriele Chianucci
