# Supplementary material for: Prey Selection by an Apex Predator: The Importance of Sampling Uncertainty
Source: PLoS One. 2012 Oct 26;7(10):e47894. doi: 10.1371/journal.pone.0047894 (PMC3482236; doi:10.1371/journal.pone.0047894)
Supplement: Contract S3 — Contract for wolf work, 2000. (PDF) [file pone.0047894.s007.pdf]

Reg. n. 3377/295
del 07.12.2000

ATTO DI CONVENZIONE

PREMESSO:

- che la presenza del lupo nel territorio provinciale è diventata, dai primi anni ottanta, continua e costante;
- che tale processo di espansione è stato però accompagnato negli ultimi anni da una recrudescenza di episodi di uccisione illegale di individui di lupo tali da mettere in pericolo la sopravvivenza della specie in molte zone;
- che la Provincia di Arezzo con Del. C.P. n. 140/97 ha istituito cinque oasi di protezione nei principali complessi forestali e montani del territorio provinciale, tra le cui finalità vi sono anche la conservazione del lupo e allo stesso tempo la riduzione dell'impatto sulle attività zootecniche attraverso il ristabilimento di un equilibrato rapporto tra predatore e prede selvatiche (cinghiale e cervidi);
- che la mancanza di conoscenze sulla consistenza, struttura e dinamica della popolazione di lupo, nonché del suo comportamento, costituisce un limite per la definizione di una valida strategia di conservazione della specie e di gestione delle oasi;
- che il Dipartimento di Etologia Ecologia ed Evoluzione dell'Università di Pisa svolge da diversi anni attività di ricerca nel Parco Nazionale delle Foreste Casentinesi, Monte Falterona e Campigna, per lo studio della presenza e delle abitudini alimentari del lupo;
- che tra la Provincia di Arezzo ed Università è stata stipulata per il triennio 1998/2000 una convenzione per una ricerca sulla popolazione di

lupo presente in provincia, che ha fornito i dati preliminari relativamente alla distribuzione della specie ed alle sue caratteristiche genetiche;

- che appare necessario proseguire la ricerca, anche per adempiere agli obblighi di monitoraggio delle specie protette di interesse comunitario previsti dalla L.R.T. 56/2000;
- che con provvedimento del Segretario Generale n. 205 del 19/10/2000 è stata approvata la convenzione tra il Dipartimento di Etologia, Ecologia ed evoluzione, dell'Università degli Studi di Pisa, la Provincia di Arezzo ed il Ministero dell'Ambiente, Servizio Conservazione della Natura, per il triennio 2001- 2003;

TRA

L'Amministrazione provinciale di Arezzo, che di seguito verrà denominata semplicemente "Provincia di Arezzo", con sede ad Arezzo in piazza della Libertà n. 3, cod. fis.: 80000610511, rappresentata dal dr. Gabriele Chianucci, Segretario Generale e Coordinatore del Servizio Caccia e Pesca,

E

Il prof. Marco Apollonio, nato a Roma il 17/11/1958, residente a Pisa - via Nicola Pisano 26, cod. fis.: PLLMRC58S17H501H nella sua qualità di professore ordinario di zoologia, nell'Università di Pisa e di Sassari;

SI CONVIENE E SI STIPULA QUANTO SEGUE:

ART. 1

La Provincia di Arezzo intende continuare lo studio triennale sulla popolazione di lupo presente nei principali complessi forestali e montani della Provincia di Arezzo, descritto all'art. 1 della convenzione tra il Dipartimento di Etologia, Ecologia ed evoluzione, dell'Università degli Studi di Pisa, la Provincia di Arezzo

ed il Ministero dell'Ambiente, Servizio Conservazione della Natura, approvata con provvedimento del Segretario Generale n. 205 del 19/10/2000, per il periodo 2001/2003;

ART. 2

Per la realizzazione del progetto la Provincia di Arezzo si impegna a:

- finanziare le spese derivanti dall'acquisizione delle strumentazioni, dei reagenti e del materiale di consumo necessari per la realizzazione dell'analisi genetica;
- finanziare le spese per la retribuzione di personale specializzato e per il trasferimento del personale operativo e di coordinamento;
- mettere a disposizione i dati in proprio possesso relativi alle presenze faunistiche all'interno delle oasi e nelle aree limitrofe, utili ai fini della ricerca;
- provvedere alla conservazione dei campioni biologici fino al momento del loro definitivo trasferimento al Dipartimento.

Il prof. Apollonio si impegna a:

- organizzare e partecipare alle operazioni di wolf-howling;
- organizzare e partecipare alle operazioni di tracciatura su neve;
- organizzare e contribuire alla raccolta di campioni biologici;
- realizzare l'analisi spettrografia dei sonogrammi ottenuti mediante il wolf-howling;
- realizzare l'analisi genetica dei campioni biologici rinvenuti nel territorio provinciale;
- elaborare i dati ottenuti mediante le tecniche sopra descritte e redigere al termine di ogni anno una relazione sullo stato di avanzamento del

progetto comprensiva dei risultati conseguiti relativamente a ciascun aspetto oggetto della ricerca. Al termine del terzo anno redigere la relazione conclusiva. I contenuti delle relazioni (annuali e finale) e le modalità della loro esposizione (cartografie, figure, etc.) saranno preventivamente concordati tra i responsabili degli Enti contraenti.

ART. 3

Il coordinamento e la responsabilità per i due enti vengono rispettivamente assegnati a:

- prof. Marco Apollonio;
- dr. Gabriele Chianucci.

Ai due responsabili è demandato il compito di concordare e garantire la realizzazione dei vari momenti operativi della ricerca nel rispetto dei tempi e delle finalità definite nel presente accordo.

ART.4 -

I costi per la realizzazione del progetto nel corso dei tre anni si prevedono in misura di:

- strumentazione per l'analisi genetica	L. 15.000.000
- reagenti e materiali di consumo	L. 15.000.000
- spese fisse e di missione	L. 30.000.000
- spese per missione di personale e consulenza	L. 30.000.000

I fondi verranno corrisposti quale contributo di ricerca con appositi provvedimenti su indicazione dei responsabili di ricerca, ai soggetti (Università, ricercatori, studenti, etc..) da essi stabiliti.

Art. 5

La proprietà dei risultati della ricerca e della relazione conclusiva dei lavori è della Provincia di Arezzo. Fermo restando il diritto d'autore, il prof. Apollonio potrà utilizzare i risultati stessi ai propri fini scientifici e didattici istituzionali. Qualora uno dei contraenti si faccia promotore e/o partecipi ad esposizioni e congressi, convegni, seminari e simili manifestazioni, nel corso delle quali si intenda esporre e far uso, sempre e soltanto a fini scientifici, dei risultati della presente convenzione sarà tenuto ad informare preventivamente l'altro contraente e comunque a citare la convenzione nel cui ambito è stata svolta la ricerca.

ART. 6

Tutte le eventuali spese di registrazione sono a carico dell'Amministrazione provinciale di Arezzo.

Letto, approvato e sottoscritto.

PROVINCIA DI AREZZO
DR. GABRIELE CHIANUCCI

PROF. MARCO APOLLONIO
