# Supplementary material for: Prey Selection by an Apex Predator: The Importance of Sampling Uncertainty
Source: PLoS One. 2012 Oct 26;7(10):e47894. doi: 10.1371/journal.pone.0047894 (PMC3482236; doi:10.1371/journal.pone.0047894)
Supplement: Contract S4 — Contract 1 for wolf work, 2001–03. (PDF) [file pone.0047894.s008.pdf]

# REGIONE TOSCANA-GIUNTA REGIONALE
## DIPARTIMENTO DELLO SVILUPPO ECONOMICO
SERVIZIO SVILUPPO AGRICOLO E RURALE CACCIA E PESCA

U. O. C. TUTELA DELLA FAUNA E GESTIONE FAUNISTICA VENATORIA

Dirigente Responsabile: Paolo Banti

| **Decreto** | **N° 3025** | del **05 Giugno 2001** |
|---|---|---|

*Pubblicità/Pubblicazione*: Atto soggetto a pubblicazione per estratto

*Allegati n°: 1*

| *Denominazione* | *Pubblicazione* | *Tipo di trasmissione* |
|---|---|---|
| A | No | Cartaceo+Digitale |

*Oggetto*:
*L.R. 3/94 - Realizzazione di un'indagine sistematica sulla "Popolazione del Lupo in Toscana". Approvazione bozza di convenzione con la Provincia di Arezzo. Impegno della spesa.*

*MOVIMENTI CONTABILI*

| *Capitolo* | *Anno* | *Tipo Movimento.* | *Numero* | *Var.* | *Data* | *Importo in Lire* | *Importo in Euro* |
|---|---|---|---|---|---|---|---|
| U-27050 | 2001 | Impegno/Assegnazione | 2170 | | 11-06-2001 | 97.500.000 | 50354,55 |

Atto soggetto al controllo interno ai sensi delle DD.G.R. nn. 372/1999, 1271/1999 e 770/2000

Controllo eseguito senza rilievi.
Atto certificato il 13-06-2001

IL DIRIGENTE

Vista la legge 11.12.92, n. 157, recante "Norme per la protezione della fauna selvatica omeoterma e per il prelievo venatorio";

Vista la legge regionale 12.01.94, n. 3 di recepimento della legge 157/92;

Visti gli artt.3, 22, 28 della L.R. n.26 del 17 marzo 2000;

Visto il decreto n. 5257 del 28/09/2000 con il quale il sottoscritto è stato nominato responsabile della struttura in oggetto;

Preso atto che in Toscana si assiste ad una diffusione in molte aree della specie Lupo, che merita ulteriori studi ed approfondimenti scientifici, anche in considerazione del suo impatto ecologico;

Considerato che la Provincia di Arezzo ha attivato fin dal 1998 una indagine sistematica sulla "Popolazione del lupo all'interno del sistema di Oasi di Protezione della Provincia di Arezzo";

Considerato che il Lupo è presente in molte zone della Toscana e che sussiste l'interesse regionale ad estendere tale ricerca per i risultati scientifici che implica;

Considerato inoltre che i risultati scientifici di tale ricerca, determinando entità, quantità e comportamenti delle popolazioni del Lupo, potranno dare utili indicazioni anche relativamente all'impatto su altre specie sia selvatiche che domestiche;

Valutato pertanto opportuno realizzare nuove azioni conoscitive, avvalendosi della Provincia di Arezzo, data l'esperienza acquisita in materia, per condurre una indagine di durata triennale sulla popolazione del lupo, finalizzandone i risultati all'intero territorio regionale;

Vista la proposta di Convenzione elaborata congiuntamente fra l'ufficio caccia della Provincia di Arezzo e la U.O.C Tutela della fauna e gestione Faunistico – Venatoria della Regione, proposta che si allega al presente atto;

Considerato altresì che la realizzazione della suddetta indagine richiede alla Regione Toscana un onere finanziario complessivo per i tre anni di durata della convenzione, di L. 195.000.000 – Euro 100709,1, da ripartirsi nel triennio 2001- 2003;

Vista la legge regionale n. 4 del 26 gennaio 2001, con la quale è stato approvato il bilancio regionale di previsione per l'esercizio finanziario 2001;

DECRETA

- realizzare una indagine sistematica, di durata triennale, sulla "Popolazione del lupo in Toscana" avvalendosi della Provincia di Arezzo, secondo quanto in narrativa descritto;

- di approvare la proposta di convenzione di durata triennale, allegata sub lettera "A", quale parte integrante e sostanziale del presento atto, con la quale vengono regolate le modalità di svolgimento, di

pagamento, le responsabilità ed i rapporti fra Regione e Provincia di Arezzo per la realizzazione dell'indagine sulla popolazione del Lupo di cui al punto precedente;

- di prevedere, per tale indagine una spesa complessiva di L. 195.000.000 – Euro 100709,1, da ripartirsi nei tre anni di durata della convenzione;

- di impegnare sul cap. 27050 del Bilancio regionale 2001, che presenta la necessaria disponibilità, la somma di L. 97.500.000 – Euro 100709,10 quale prima annualità da assegnare alla Provincia di Arezzo per lo svolgimento della indagine citata;

- di rinviare l'assunzione degli impegni, relativi alla seconda annualità ed al saldo finale, sui competenti capitoli del bilancio regionale 2002 e 2003 a successivi atti di impegno;

- la liquidazione, secondo le modalità previste dall'art. 113 della L.R. 28/77, delle somme sull'impegno assunto con il presente atto, avverrà entro tre mesi dalla stipula della convenzione, dietro presentazione, da parte della Provincia di Arezzo di relazione di inizio dei lavori di indagine, corredata dal progetto definitivo dell'indagine stessa;

Il presente provvedimento, soggetto a pubblicità ai sensi della L.R. 9/95 in quanto conclusivo del procedimento amministrativo regionale, è pubblicato per estratto sul Bollettino Ufficiale della Regione Toscana ai sensi dell'art. 3, comma 2, della L.R. 18/96.

MB/cp

Il Dirigente
PAOLO BANTI
