# Supplementary material for: Prey Selection by an Apex Predator: The Importance of Sampling Uncertainty
Source: PLoS One. 2012 Oct 26;7(10):e47894. doi: 10.1371/journal.pone.0047894 (PMC3482236; doi:10.1371/journal.pone.0047894)
Supplement: Contract S5 — Contract 2 for wolf work, 2001–03. (PDF) [file pone.0047894.s009.pdf]

CONVENZIONE TRA IL DIPARTIMENTO DI ETOLOGIA , ECOLOGIA ED EVOLUZIONE, DELL' UNIVERSITA DEGLI STUDI DI PISA, LA PROVINCIA DI AREZZO ED IL MINISTERO DELL'AMBIENTE, SERVIZIO CONSERVAZIONE DELLA NATURA

PREMESSO:

- che la presenza del lupo nel territorio provinciale è diventata, dai primi anni ottanta, continua e costante
- che tale processo di espansione è stato però accompagnato negli ultimi anni da una recrudescenza di episodi di uccisione illegale di individui di lupo tali da mettere in pericolo la sopravvivenza della specie in molte zone
- che la Provincia di Arezzo con Del. C.P. n° 140/97 ha istituito cinque oasi di protezione nei principali complessi forestali e montani del territorio provinciale, tra le cui finalità vi sono anche la conservazione del lupo e allo stesso tempo la riduzione dell'impatto sulle attività zootecniche attraverso il ristabilimento di un equilibrato rapporto tra predatore e prede selvatiche (cinghiale e cervidi)
- che la raccolta di conoscenze sulla consistenza, struttura e dinamica della popolazione di lupo, nonché sul suo comportamento, costituisce un presupposto necessario per la definizione di una valida strategia di conservazione della specie e di gestione delle oasi
- che il Dipartimento di Etologia Ecologia ed Evoluzione dell'Università di Pisa svolge da diversi anni attività di ricerca/nel Parco Nazionale delle Foreste Casentinesi, Monte Falterona e Campigna, per lo studio della presenza e delle abitudini alimentari del lupo
- che tra Provincia ed Università è stata stipulata per il triennio 1998-2000 una convenzione per una ricerca sulla popolazione di lupo presente in provincia, che ha fornito i dati preliminari relativamente alla distribuzione della specie ed alle sue caratteristiche genetiche
- che appare necessario proseguire la ricerca, anche per adempiere agli obblighi di monitoraggio delle specie protette di interesse comunitario previsti dalla LRT 56/2000

TRA

L' Amministrazione Provinciale di Arezzo, che di seguito verrà denominata "Provincia", con sede ad Arezzo in Piazza della Libertà 3, codice fiscale 80000610511 , rappresentata dal Segretario Generale Dr. Gabriele Chianucci

Il Dipartimento di Etologia, Ecologia ed Evoluzione Università degli Studi di Pisa che di seguito verrà denominato "Dipartimento", con sede a Pisa in via Alessandro Volta n. 6, codice fiscale 80003670504, rappresentato dal suo Direttore Prof. Fernando Dini

Il Ministero dell'Ambiente, Servizio per la conservazione della natura, che di seguito verrà denominato "Ministero" con sede in Roma Via Capitan Bavastro 174, rappresentato dal Dirigente della Divisione II Dr. Alessandro La Posta

SI CONVIENE E STIPULA QUANTO SEGUE:

art. 1

Ministero, Dipartimento e Provincia intendono proseguire per il triennio 2001-2003, il primo studio triennale 1998-2000 sulla popolazione di lupo presente nei principali complessi forestali e montani della Provincia di Arezzo, avente le seguenti finalità:

a) monitorare la presenza del lupo nelle diverse aree del comprensorio
b) determinare la consistenza minima della popolazione su base annuale
c) verificare la presenza di attività riproduttiva su base annuale
d) valutare la struttura e la dinamica della popolazione
e) ottenere indicazioni sull'entità degli spostamenti individuali e/o del branco
f) valutare il livello di imparentamento tra i branchi
g) valutare entità, distribuzione temporale e localizzazione dei danni subiti dalle attività zootecniche
h) valutare le densità delle popolazioni di ungulati selvatici.

I dati ottenuti permetteranno di tracciare un quadro della popolazione di lupi presente nel territorio provinciale con riferimento al numero, struttura e stabilità dei branchi esistenti, ai loro rapporti di parentela, alla stabilità della loro presenza nelle diverse aree, alla natura spaziale e temporale degli spostamenti, al rapporto con le popolazioni di ungulati selvatici e all'impatto sulle attività zootecniche.
Tali informazioni, allo stato attuale mai raccolte in Italia su scala provinciale, costituiranno l'indispensabile supporto per la definizione delle linee di gestione che l'Amministrazione Provinciale di Arezzo dovrà nei prossimi anni sviluppare riguardo a due cruciali aspetti:

1. Gestione delle oasi di protezione; le Oasi di protezione costituiscono uno degli obiettivi prioritari della programmazione faunistica della provincia di Arezzo, Il successo nella loro gestione futura dipenderà dalla capacità di prevedere e risolvere alcune problematiche che potranno essere connesse alla loro costituzione. Tra queste si può includere la dinamica delle popolazioni di ungulati, in particolare del cinghiale, ed il contenimento, entro livelli accettabili, dei danni causati dal lupo alle attività zootecniche. I risultati della ricerca potranno suggerire soluzioni a tali problematiche ed integrazioni all'attività di pianificazione faunistico-venatoria fino ad oggi attuata dalla Provincia di Arezzo.
2. Protezione e conservazione del lupo in Provincia di Arezzo; la ricerca fornirà un supporto indispensabile per valutare l'entità delle perdite di soggetti di lupo dovute ai vari fattori di mortalità accidentale o illegale ed il loro impatto sulla conservazione a medio termine della popolazione di lupo che gravita nel territorio provinciale.

La ricerca verrà condotta attraverso l'utilizzo dei seguenti metodi:

Wolf-howling e analisi spettrografica dei sonogrammi ottenuti dalla registrazione delle risposte
Tracciatura su neve dei branchi individuati
Analisi del DNA nucleare
Censimento delle popolazioni di ungulati selvatici e domestici.

Il dettaglio relativo a ciascuno dei metodi di ricerca è contenuto nel documento allegato alla presente convenzione, elaborato dal Prof. Marco Apollonio.

Art. 2

PER LA REALIZZAZIONE DEL PROGETTO

La Provincia si impegna a:

- finanziare con apposito atto tra Provincia e Dipartimento le spese di base necessarie per lo svolgimento della ricerca;
- mettere a disposizione i dati in proprio possesso relativi alle presenze faunistiche all'interno delle oasi e nelle aree ad esse limitrofe, utili ai fini della ricerca;

- provvedere alla conservazione dei campioni biologici fino al momento del loro definitivo trasferimento al Dipartimento.

Il Dipartimento si impegna a:
- organizzare e partecipare alle operazioni di wolf-howling;
- organizzare e partecipare alle operazioni di tracciatura su neve;
- organizzare e contribuire alla raccolta di campioni biologici;
- realizzare l'analisi spettrografica dei sonogrammi ottenuti mediante il wolf-howling;
- realizzare l'analisi genetica dei campioni biologici rinvenuti nel territorio provinciale;
- elaborare i dati ottenuti mediante le tecniche sopra descritte e redigere al termine di ogni anno una relazione sullo stato di avanzamento del progetto comprensiva dei risultati conseguiti relativamente a ciascun aspetto oggetto della ricerca. Al termine del terzo anno redigere la relazione conclusiva. I contenuti delle relazioni (annuali e finale) e le modalità della loro esposizione (cartografie, figure, etc.) saranno preventivamente concordati tra i responsabili degli Enti contraenti.

Il Ministero si impegna a:
- dare il proprio patrocinio alla ricerca;
- collaborare con le proprie informazioni e dati allo svolgimento della ricerca;

art. 3

Il coordinamento e la responsabilità per gli Enti vengono rispettivamente assegnati a:
- per il Dipartimento: Prof. Marco APOLLONIO – del Dipartimento di Etologia, Ecologia ed Evoluzione dell' Università di Pisa;
- per la Provincia di Arezzo: Dr. Gabriele CHIANUCCI – Segretario generale della provincia di Arezzo;
- per il Ministero: Dr. Alessandro LA POSTA – Dirigente della Divisione II del Servizio Conservazione della natura.

Ai responsabili è demandato il compito di concordare e garantire la realizzazione dei vari momenti operativi della ricerca nel rispetto dei tempi e delle finalità definite nel presente accordo.

Art. 4

RISULTATI DELLA RICERCA

La Proprietà dei risultati della ricerca e della relazione conclusiva dei lavori è della provincia di Arezzo. Fermo restando il diritto d'autore, il Dipartimento potrà utilizzare i risultati stessi ai propri fini scientifici e didattici istituzionali.
Qualora uno dei contraenti si faccia promotore e/o partecipi ad esposizioni e congressi, convegni, seminari e simili manifestazioni, nel corso delle quali si intenda esporre e far uso, sempre e soltanto a fini scientifici, dei risultati della presente convenzione sarà tenuto ad informare preventivamente l'altro contraente e comunque a citare la convenzione nel cui ambito è stata svolta la ricerca.

Art. 5

DISPOSIZIONI FINALI

Tutte le eventuali spese di registrazione sono a carico dell'Amministrazione Provinciale di Arezzo.

- 5 OTT. 2000

Per il Ministero
Dr. Alessandro LA POSTA

Per la Provincia di Arezzo
Dr. Gabriele CHIANUCCI

Per il Dipartimento
Prof. Ferdinando DINI

Università di Pisa – Dip. Etologia, Ecologia, Evoluzione
