# Supplementary material for: Prey Selection by an Apex Predator: The Importance of Sampling Uncertainty
Source: PLoS One. 2012 Oct 26;7(10):e47894. doi: 10.1371/journal.pone.0047894 (PMC3482236; doi:10.1371/journal.pone.0047894)
Supplement: Contract S6 — Contract for wolf work, 2004. (PDF) [file pone.0047894.s010.pdf]

Provincia di Arezzo

Prot. n. PG 23094 /42-23-27

Arezzo, lì 25 MAG. 2004

Al Prof. Marco Apollonio
Dip.to di Zoologia ed Antropologia Biologica
Università degli studi di Sassari
Via Muroni 25
SASSARI

Oggetto: Convenzione progetto lupo

Illustr.mo Professore,

come da precedenti accordi, Le invio copia della convenzione stipulata tra la Provincia di Arezzo ed il Dip.to di Zoologia ed Antropologia Biologica dell' Università degli studi di Sassari per lo svolgimento di un progetto di ricerca sul lupo nella Provincia di Arezzo.

Nei prossimi giorni sarà nostra cura provvedere al trasferimento dei fondi necessari allo svolgimento del primo anno di ricerca.

Il Coordinatore
Dott. Gabriele Chianucci

PAGAMENTO
[illegible]

Reg. n. 5541/257

ATTO DI CONVENZIONE

**PREMESSO:**

- che la presenza del lupo nel territorio provinciale è diventata, dai primi anni ottanta, continua e costante
- che tale processo di espansione si sta osservando anche in nuove aree dove prima la specie era assente con importanti ripercussioni per la pianificazione faunistico-venatoria della Provincia di Arezzo e per la gestione dEll'impatto sulle attività zootecniche;
- che tale processo è stato però accompagnato da una recrudescenza di episodi di uccisione illegale di individui di lupo tali da mettere in pericolo la sopravvivenza della specie in molte zone
- che la Provincia di Arezzo con Del. C.P. n° 140/97 ha istituito cinque oasi di protezione nei principali complessi forestali e montani del territorio provinciale, tra le cui finalità vi sono anche la conservazione del lupo e allo stesso tempo la riduzione dell'impatto sulle attività zootecniche attraverso il ristabilimento di un equilibrato rapporto tra predatore e prede selvatiche ( cinghiale e cervidi)
- che la raccolta di conoscenze sulla consistenza, struttura e dinamica di popolazione (natalità, mortalità, dispersione) del lupo costituisce un presupposto necessario per la definizione di una valida strategia di conservazione della specie e di gestione delle oasi e dell'intero territorio provinciale;

- che il Prof. Marco Apollonio del Dipartimento di Zoologia e Antropologia biologica dell'Università degli Studi di Sassari HA SVOLTO PER MOLTI anni attività di ricerca/nel Parco Nazionale delle Foreste Casentinesi, Monte Falterona e Campigna, per lo studio della presenza e delle abitudini alimentari del lupo

- che tra Provincia ed il Prof. Marco Apollonio è stata stipulata per il triennio 2000-2003 una convenzione per una ricerca sulla popolazione di lupo presente in provincia, che ha fornito i dati preliminari relativamente alla distribuzione della specie ed alle sue caratteristiche genetiche

- che appare necessario proseguire la ricerca, anche per adempiere agli obblighi di monitoraggio delle specie protette di interesse comunitario previsti dalla LRT 56/2000

**TRA**

**L' Amministrazione Provinciale di Arezzo, che di seguito verrà denominata "Provincia", con sede ad Arezzo in Piazza della Libertà 3, codice fiscale 80000610511 , rappresentata dal Segretario Generale Dr. Gabriele Chianucci**

**IL Dipartimento di Zoologia e Antropologia biologica dell'Università degli Studi di Sassari , che di seguito verrà denominato "Dipartimento", con sede in Sassari in via Muroni n° 25, codice fiscale 00196350904,rappresentato amministrativamente dal suo Direttore Prof. Achille Casale.**

SI CONVIENE E STIPULA QUANTO SEGUE:

art. 1

Provincia e Dipartimento intendono proseguire per il quinquennio 2004-2008, lo studio triennale 2000-2003 sulla popolazione di lupo presente nei principali complessi forestali e montani della Provincia di Arezzo, con particolare riferimento all'area dell' Alpe di Catenaia, con le seguenti finalità:

a) monitorare la presenza del lupo nelle diverse aree del comprensorio
b) determinare la consistenza minima della popolazione su base annuale
c) verificare la presenza di attività riproduttiva su base annuale
d) valutare la struttura e la dinamica della popolazione
e) valutare il livello di imparentamento tra i branchi
f) monitorare il processo di colonizzazione di nuove aree
g) valutare mortalità e dispersione di soggetti di lupo mediante tecniche radiotelemetriche
h) approfondire le relazioni tra lupo ed ungulati selvatici nel complesso dell' Alpe di Catenaia

I dati ottenuti permetteranno di tracciare un quadro generale sulla popolazione di lupi presente nel territorio provinciale con riferimento al numero, struttura e stabilità dei branchi esistenti, ai loro rapporti di parentela, alla stabilità della loro presenza nelle diverse aree, alla' entità dei processi di colonizzazione di nuove aree. Le tecniche telemetriche e lo studio intensivo nel complesso dell' Alpe di Catenaia forniranno dati più precisi sui fattori che regolano la dinamica

di popolazione del lupo, sui suoi spostamenti, sull'impatto sulle popolazioni di ungulati selvatici e domestici.

Tali informazioni, allo stato attuale mai raccolte in Italia su scala provinciale, costituiranno l'indispensabile supporto per la definizione delle linee di gestione che l'Amministrazione Provinciale di Arezzo dovrà nei prossimi anni sviluppare riguardo a due cruciali aspetti:

1. Gestione delle oasi di protezione; le Oasi di protezione costituiscono uno degli obiettivi prioritari della programmazione faunistica della provincia di Arezzo, Il successo nella loro gestione futura dipenderà dalla capacità di prevedere e risolvere alcune problematiche che potranno essere connesse alla loro costituzione. Tra queste si può includere la dinamica delle popolazioni di ungulati, in particolare del cinghiale, ed il contenimento, entro livelli accettabili, dei danni causati dal lupo alle attività zootecniche. I risultati della ricerca potranno suggerire soluzioni a tali problematiche ed integrazioni all'attività di pianificazione faunistico-venatoria fino ad oggi attuata dalla Provincia di Arezzo.
2. Protezione e conservazione del lupo in Provincia di Arezzo; la ricerca fornirà un supporto indispensabile per valutare l'entità delle perdite di soggetti di lupo dovute ai vari fattori di mortalità accidentale o illegale ed il loro impatto sulla conservazione a medio termine della popolazione di lupo che gravita nel territorio provinciale.

La ricerca verrà condotta attraverso l'utilizzo dei seguenti metodi:

Wolf-howling e analisi spettrografica dei sonogrammi ottenuti dalla registrazione delle risposte

Tracciatura su neve dei branchi individuati

Analisi genetica non invasiva del DNA nucleare

Cattura e monitoraggio radiotelemetrico di alcuni soggetti di lupo

**Art. 2**

PER LA REALIZZAZIONE DEL PROGETTO

La Provincia si impegna a:

- finanziare con apposito atto tra Provincia e Dipartimento le spese di base necessarie per lo svolgimento della ricerca sotto forma di contributo di ricerca, per un importo totale di € 80.000,00 per i cinque anni del progetto;
- mettere a disposizione i dati in proprio possesso relativi alle presenze faunistiche all'interno delle oasi e nelle aree ad esse limitrofe, utili ai fini della ricerca;
- provvedere alla conservazione dei campioni biologici fino al momento del loro definitivo trasferimento al Dipartimento.

Il Dipartimento si impegna a:

- organizzare e partecipare alle operazioni di wolf-howling;
- organizzare e partecipare alle operazioni di tracciatura su neve;
- organizzare e contribuire alla raccolta di campioni biologici;
- organizzare e realizzare le operazioni di cattura di alcuni soggetti di lupo;

- realizzare l'analisi spettrografica dei sonogrammi ottenuti mediante il wolf-howling;
- realizzare l'analisi genetica dei campioni biologici rinvenuti nel territorio provinciale;
- elaborare i dati ottenuti mediante le tecniche sopra descritte e redigere al termine di ogni anno una relazione sullo stato di avanzamento del progetto comprensiva dei risultati conseguiti relativamente a ciascun aspetto oggetto della ricerca. Al termine del terzo anno redigere la relazione conclusiva. I contenuti delle relazioni (annuali e finale) e le modalità della loro esposizione (cartografie, figure, etc.) saranno preventivamente concordati tra i responsabili degli Enti contraenti.

art. 3

COORDINAMENTO

Il coordinamento e la responsabilità per gli Enti vengono rispettivamente assegnati a:

- per il Dipartimento: Prof. Marco APOLLONIO - Professore Ordinario di zoologia dei vertebrati, dell' Università di Sassari; per la Provincia di Arezzo: Dr. Gabriele CHIANUCCI – Segretario generale della provincia di Arezzo;

Ai responsabili è demandato il compito di concordare e garantire la realizzazione dei vari momenti operativi della ricerca nel rispetto dei tempi e delle finalità definite nel presente accordo.

Art. 4

RISULTATI DELLA RICERCA

La Proprietà dei risultati della ricerca e della relazione conclusiva dei lavori è della provincia di Arezzo. Fermo restando il diritto d'autore, il Dipartimento potrà utilizzare i risultati stessi ai propri fini scientifici e didattici istituzionali.

Qualora uno dei contraenti si faccia promotore e/o partecipi ad esposizioni e congressi, convegni, seminari e simili manifestazioni, nel corso delle quali si intenda esporre e far uso, sempre e soltanto a fini scientifici, dei risultati della presente convenzione sarà tenuto ad informare preventivamente l'altro contraente e comunque a citare la convenzione nel cui ambito è stata svolta la ricerca.

Art. 5

DISPOSIZIONI FINALI

Tutte le eventuali spese di registrazione sono a carico dell'Amministrazione Provinciale di Arezzo.

Per L'Amministrazione Provinciale di Arezzo
Il Segretario Generale Dr. Gabriele Chianucci

---------------------------------------------------------

Per il Dip.to di Zoologia e Antropologia Biologica dell'Università
degli Studi di Sassari
Il Responsabile Amministrativo Prof. Achille Casale

---------------------------------------------------------

Arezzo..... lì 22.04.2004
