# Supplementary material for: Prey Selection by an Apex Predator: The Importance of Sampling Uncertainty
Source: PLoS One. 2012 Oct 26;7(10):e47894. doi: 10.1371/journal.pone.0047894 (PMC3482236; doi:10.1371/journal.pone.0047894)
Supplement: Contract S7 — Contract for wolf work, 2005–07. (PDF) [file pone.0047894.s011.pdf]

REGIONE TOSCANA-GIUNTA REGIONALE

DIREZIONE GENERALE SVILUPPO ECONOMICO

AREA DI COORDINAMENTO POLITICHE PER LO SVILUPPO RURALE

SETTORE TUTELA DELLA FAUNA E GESTIONE FAUNISTICO VENATORIA, MECCANIZZAZIONE AGRICOLA, CALAMITA' NATURALI, SISTEMI IRRIGUI

Dirigente Responsabile: Paolo Banti

**Decreto**	**N° 2342**	del **26 Aprile 2005**

Pubblicità/Pubblicazione: Atto soggetto a pubblicazione per estratto

Allegati n°: 0

Oggetto:
Contributo alla Provincia di Arezzo per la realizzazione di un progetto di ricerca faunistica di interesse regionale sulla popolazione del lupo

MOVIMENTI CONTABILI

Capitolo	*Anno*	*Tipo Movimento.*	*Numero*	*Var.*	*Data*	*Importo in Euro*
U-27051	2005	Impegno/Assegnazione	2324		02-05-2005	34.400,00
U-27051	2006	Impegno/Assegnazione	2324		02-05-2005	33.200,00
U-27051	2007	Impegno/Assegnazione	2324		02-05-2005	12.400,00

Atto soggetto al controllo interno ai sensi della D.G.R. n. 1315/2003

Controllo eseguito senza rilievi.
Atto certificato il 05-05-2005

IL DIRIGENTE

Visto l'articolo 3 della legge regionale 17 marzo 2000 n.26 "Riordino della legislazione regionale in materia di organizzazione personale" e successive modifiche ed integrazioni;

Vista la legge regionale 5 agosto 2003 , n. 44 " Ordinamento della dirigenza e della struttura operativa della Regione. Modifiche alla Legge Regionale 17 marzo 2000, n. 26, ed in particolare l'art. 8 che definisce le competenze del responsabile dei settore;

Visto il decreto del Direttore Generale dello Sviluppo Economico n. 1738 del 04/04/2005, con il quale il sottoscritto è stato nominato responsabile del settore " Tutela della fauna e gestione faunistico venatoria, meccanizzazione agricola, calamità naturali, sistemi irrigui";

Vista la legge 11 febbraio 1992 n. 157 recante "Norme per la protezione della fauna selvatica omeoterma e per il prelievo venatorio"che definisce la fauna selvatica patrimonio indisponibile dello stato affidandone la gestione alle regioni;

Vista la legge regionale 12 gennaio 1994 n. 3 di recepimento della legge 157/1992;

Visto il capitolo 27051 del bilancio che prevede un fondo a disposizione della Giunta Regionale per iniziative di interesse regionale in favore dell'ambiente e della fauna, per attività di educazione e propaganda, nonché per eventuali contributi ad enti o associazioni operanti nel settore per l'espletamento dei compiti propri della Giunta Regionale;

Vista la nota prot.1207/15.2 del 20/01/2005 con la quale la Provincia di Arezzo intende proseguire il progetto triennale di ricerca faunistica di interesse regionale sulla popolazione del lupo e degli ungulati selvatici in Alpe di Catenaria ;

Valutata positivamente tale iniziativa e ritenuto che la stessa risponde all'interesse generale di tutela e conservazione della fauna e che i dati e le informazioni emerse oltre a confluire nell'aggiornamento del Piano Faunistico Venatorio provinciale , consentono una migliore gestione delle oasi di protezione faunistica ;

Richiamata la deliberazione della Giunta regionale n. 674 del 03.09.1992 di determinazione dei criteri e delle modalità di cui all'articolo 12 della Legge 241/90 per la concessione di contributi, sussidi ed ausili finanziari a persone, Enti pubblici e privati;

Considerato che nella suddetta deliberazione è previsto che per iniziative di carattere regionale può essere erogato un contributo fino ad un massimo del 80% della spesa prevista;

Ritenuto concedere un contributo complessivo di Euro 80.000,00 per la realizzazione dell'iniziativa pari all'80% dell'importo totale previsto pari a 100.000,00 euro, importo ritenuto congruo in rapporto alle attività previste;

Ritenuto di ripartire il contributo complessivo di euro 80.000,00 nel triennio 2005-2007 secondo il piano economico agli atti di questo ufficio, presentato dal soggetto beneficiario, che prevede un contributo per il

primo anno di euro 34.400,00 (pari all’80% del fabbisogno stimato in euro 43.000,00) , di euro 33.200,00 per il secondo anno (pari all’80% del fabbisogno stimato in euro 41.500,00) e di euro 12.400,00 per il terzo anno (pari all’80% del fabbisogno stimato in euro 15.500,00);

Vista la Legge regionale n. 72 del 20/12/2004 di approvazione del Bilancio di Previsione per l’anno 2005 e del bilancio pluriennale 2005-2007;

Vista la Delibera di Giunta n. 1385 del 27/12/2004 “ Approvazione Bilancio Gestionale 2005 e pluriennale 2005/2007 “;

Considerato che il contributo non è soggetto a ritenuta d'acconto ai sensi dell'articolo 26 del DPR 600/73 in quanto trattasi di Ente non a scopo di lucro;

DECRETA

- di concedere alla Provincia di Arezzo, per i motivi di cui in premessa, un contributo per la realizzazione del progetto di ricerca faunistica di interesse regionale sulla popolazione del lupo di Euro 80.000,00 pari all’80% dell’importo complessivo stimato in Euro 100.000,00;

- di assumere sul capitolo 27051 del bilancio di previsione dell’esercizio corrente, che presenta la necessaria disponibilità, l’impegno di Euro 34.400,00 (pari all’80% del fabbisogno stimato in euro 43.000,00) a favore della Provincia di Arezzo , importo corrispondente al contributo previsto per il primo anno di attività; sul bilancio pluriennale 2006 la somma di euro 33.200,00 e sul bilancio pluriennale 2007 la somma di euro 12.400,00;

- di richiedere al soggetto beneficiario la rendicontazione delle spese sostenute entro il 31.12.2005;

- di liquidare la somma impegnata con il presente atto, secondo le modalità previste dall’art. 44 e 45 DPGR n. 61/r del 19 dicembre 2001, dietro presentazione di regolare rendicontazione;

- di procedere all’iscrizione del suddetto contributo nell’elenco dei beneficiari ai sensi del DPR 118/2000;

Il presente provvedimento, soggetto a pubblicità ai sensi della legge regionale 9/95 in quanto atto conclusivo di procedimento amministrativo regionale, è pubblicato per estratto sul Bollettino Ufficiale della Regione Toscana ai sensi dell'articolo 3, comma 2 della legge regionale 18/96 e successive modifiche ed integrazioni.

Il Dirigente

PAOLO BANTI
