# Supplementary material for: Prey Selection by an Apex Predator: The Importance of Sampling Uncertainty
Source: PLoS One. 2012 Oct 26;7(10):e47894. doi: 10.1371/journal.pone.0047894 (PMC3482236; doi:10.1371/journal.pone.0047894)
Supplement: Contract S8 — Contract for wolf work, 2008–09. (PDF) [file pone.0047894.s012.pdf]

REGIONE TOSCANA-GIUNTA REGIONALE

DIREZIONE GENERALE SVILUPPO ECONOMICO

AREA DI COORDINAMENTO POLITICHE PER LO SVILUPPO RURALE

SETTORE FAUNISTICO VENATORIO, PESCA DILETTANTISTICA, SERVIZI ALLE IMPRESE AGRICOLE

Il Dirigente Responsabile/ Il Responsabile di P.O. delegato: Paolo Banti

**Decreto**	**N° 1850**	del **17 Aprile 2008**

Pubblicità/Pubblicazione: Atto soggetto a pubblicazione su Banca Dati (PBD)

Allegati n°: 1

Denominazione	*Pubblicazione*	*Tipo di trasmissione*
A	No	Cartaceo

Oggetto:
L.R. 3/94 - Contributo straordinario alla Provincia di Arezzo per la realizzazione di un progetto biennale di ricerca faunistica di interesse regionale sulla popolazione del lupo. Impegno della spesa.

MOVIMENTI CONTABILI

Capitolo	*Anno*	*Tipo Movimento.*	*Numero*	*Var.*	*Data*	*Importo*	*Cod. Gest.*
U-55011	2008	Impegno/Assegnazione	2179		28-04-2008	30.000,00	153200
U-55011	2009	Impegno/Assegnazione	2179		28-04-2008	30.000,00	153200

Atto soggetto al controllo interno ai sensi della D.G.R. n. 1315/2003 e della D.G.R. n. 506/2006

Controllo eseguito senza rilievi.
Atto certificato il 07-05-2008

Errata Corrige:

- Al punto 13 narrativa, DOPO "biennio 2008/2009 di" DELE "euro 72.000,00)" e ADDE "euro 75.000,00"

IL DIRIGENTE

Vista la legge 11 febbraio 1992, n. 157 “Norme per la protezione della fauna selvatica omeoterma e per il prelievo venatorio”;

Vista la legge regionale 12 gennaio 1994, n. 3 di recepimento della legge 157/1992;

Visto l’art.3 della L.R 17 marzo 2000, n.26 e successive modifiche ed integrazioni;

Vista la L.R. 5 Agosto 2003, n. 44 ed in particolare l’art. 8;

Visto il decreto n. 89 del 16/01/2006 con il quale il sottoscritto è stato nominato responsabile del Settore “Faunistico venatorio, Pesca Dilettantistica, Servizi alle Imprese agricole”;

Vista la richiesta della Provincia di Arezzo, che si allega in copia sub lettera “A”, di un contributo straordinario finalizzato alla prosecuzione, anche per il biennio 2008 – 2009, del progetto di ricerca faunistica di interesse regionale sulla popolazione del lupo;

Rilevato che con decreto n. 2342 del 26 aprile 2005, la Regione Toscana provvedeva ad affidare alla Provincia di Arezzo una indagine sistematica sulla popolazione del Lupo in Toscana per il triennio 2005 - 2007;

Considerato che la Provincia di Arezzo ha attivato fin dal 1998 una indagine sistematica sulla “Popolazione del lupo all’interno del sistema di Oasi di Protezione della Provincia di Arezzo”;

Considerato che il Lupo è presente in molte zone della Toscana e che sussiste l’interesse regionale ad estendere tale ricerca per i risultati scientifici che implica;

Preso atto che in Toscana si assiste ad una diffusione in molte aree della specie Lupo, che merita ulteriori studi ed approfondimenti scientifici, anche in considerazione del suo impatto ecologico;

Considerato inoltre che i risultati scientifici di tale ricerca, determinando entità, quantità e comportamenti delle popolazioni del Lupo, potranno dare utili indicazioni anche relativamente all’impatto su altre specie sia selvatiche che domestiche;

Valutato pertanto opportuno realizzare nuove azioni conoscitive, affidando alla Provincia di Arezzo, data l’esperienza acquisita in materia, l’incarico di condurre una indagine di durata biennale sulla popolazione del lupo, finalizzandone i risultati all’intero territorio regionale;

Considerato che la realizzazione della suddetta indagine ha un costo totale previsto per il biennio 2008/2009 di € 72.000,00 con un onere finanziario per la Regione Toscana di complessivi € 60.000,00 ripartiti in € 30.000,00 per l’anno 2008 ed € 30.000,00 per l’anno 2009;

Richiamata la deliberazione della Giunta regionale n. 674 del 03.09.1992 di determinazione dei criteri e delle modalità di cui all'articolo 12 della Legge 241/90 per la concessione di contributi, sussidi ed ausili finanziari a persone, Enti pubblici e privati;

Valutato congruo l'impegno di spesa previsto;

Considerato che il contributo non è soggetto a ritenuta d'acconto ai sensi dell'articolo 26 del DPR 600/73 in quanto trattasi di Ente non a scopo di lucro;
Dato atto dell'iscrizione dei contributi erogati nell'elenco dei beneficiari ai sensi del D.P.R. n. 118 del 7/4/2000;

Vista la L.R. n. 68 del 21/12/2007, con la quale è stato approvato il Bilancio regionale di previsione per l'esercizio finanziario 2008 ed il Bilancio pluriennale 2008/2010;

Vista la Deliberazione G.R. n.996 del 27/12/2007 di approvazione del Bilancio gestionale 2008 e pluriennale 2008/2010;

DECRETA

- di approvare, per i motivi in narrativa esposti, la richiesta della Provincia di Arezzo di prosecuzione per il biennio 2008/2009 dell'indagine sulla popolazione del lupo in toscana;

- di impegnare sul capitolo 55011 del Bilancio regionale 2008, che presenta la necessaria disponibilità, la somma di Euro 30.000,00, a favore della Provincia di Arezzo, quale contributo straordinario da erogare per lo svolgimento dell'annualità 2008 dell'indagine citata;

- di impegnare sul capitolo 55011 del Bilancio Pluriennale 2009, che presenta la necessaria disponibilità, la somma di Euro 30.000,00 a favore della Provincia di Arezzo, quale contributo straordinario da erogare per lo svolgimento dell'annualità 2009 dell'indagine citata;

- di provvedere, secondo le modalità previste dall'articolo 44 del DPGR n. 61/r del 19/12/2001, alla liquidazione per ogni annualità del primo 50% del contributo assegnato, dietro presentazione di relazione di inizio attività relativa all'annualità medesima;

- di provvedere, secondo le modalità previste dall'articolo 44 del DPGR n. 61/r del 19/12/2001, a liquidare per ogni annualità il restante 50% del contributo assegnato, previa presentazione del rendiconto finale delle spese sostenute nell'annualità medesima imputandone la spesa all'impegno assunto con il presente atto;

- di procedere all'iscrizione del suddetto contributo nell'elenco dei beneficiari ai sensi del DPR 118/2000;

- di dichiarare i suddetti contributi soggetti a rendicontazione, ai sensi dell'art. 158 del d.lgs 267/2000;

Il presente atto, soggetto a pubblicazione ai sensi dell'art. 18, comma 2, lett. A) della L.R. 23/2007, in quanto conclusivo del procedimento amministrativo regionale, è pubblicato integralmente sulla banca dati degli atti amministrativi della Giunta regionale.

Il Dirigente

PAOLO BANTI
