# Supplementary material for: Prey Selection by an Apex Predator: The Importance of Sampling Uncertainty
Source: PLoS One. 2012 Oct 26;7(10):e47894. doi: 10.1371/journal.pone.0047894 (PMC3482236; doi:10.1371/journal.pone.0047894)
Supplement: Contract S9 — Contract for wolf work, 2009–2013. (PDF) [file pone.0047894.s013.pdf]

REG. 9064/509
DEL 11.08.2009

ATTO DI CONVENZIONE

Premesso:

- Che la presenza del lupo nel territorio provinciale è diventata, dai primi anni ottanta, continua e costante;
- Che tale processo di espansione si sta osservando anche in nuove aree dove prima la specie era assente con importanti ripercussioni per la pianificazione faunistico-venatoria della provincia di Arezzo e per la gestione dell'impatto sulle attività zootecniche ;
- Che tale processo è stato però accompagnato da una recrudescenza di episodi di uccisione illegale di individui di lupo tali da mettere in pericolo la sopravvivenza della specie in molte zone;
- Che la provincia di Arezzo ha istituito cinque oasi di protezione nei principali complessi forestali e montani del territorio provinciale, tra le cui finalità vi sono anche la conservazione del lupo e allo stesso tempo la riduzione dell'impatto sulle attività zootecniche attraverso il ristabilimento di un equilibrato rapporto tra predatore e prede selvatiche (cinghiale e cervidi);
- Che la raccolta di conoscenze sulla consistenza, struttura e dinamica di popolazione (natalità, mortalità, dispersione) del lupo costituisce un presupposto necessario per la definizione di una valida strategia di conservazione della specie e di gestione delle oasi e dell'intero territorio provinciale;
- Che il prof. Marco Apollonio del dipartimento di Zoologia e Genetica Evoluzionistica dell'Università degli studi di Sassari ha svolto per molti anni attività di ricerca nel Parco Nazionale delle Foreste Casentinesi, Monte Falterona e Campigna, per lo studio della presenza e delle abitudini del lupo;
- Che tra la provincia di Arezzo e il Prof. Marco Apollonio sono state stipulate fino al 2008 convenzioni per effettuare ricerche sulla popolazione lupo presente in Provincia, fornendo dati importanti relativamente alla distribuzione della specie ed alle sue caratteristiche genetiche;
- Che appare necessario proseguire la ricerca, per adempiere agli obblighi di monitoraggio delle specie protette di interesse comunitario previsti dalla LRT 56/2000;

TRA

L'Amministrazione Provinciale di Arezzo, che di seguito verrà denominata "Provincia", con sede ad Arezzo in Piazza della Libertà 3, codice fiscale 80000610511, rappresentata dal segretario Generale Dott. Gabriele Chianucci;

L'Ambito Territoriale di Caccia AR3, che di seguito verrà denominato "ATC3", con sede ad Arezzo Via Don Luigi Sturzo, 43/F, codice fiscale 92023320515, rappresentata dal Presidente Giorgio Kwiatkoski";

Il Dipartimento di Zoologia e Genetica Evoluzionistica dell'Università degli Studi di Sassari, che di seguito verrà denominato "Dipartimento" con sede in Sassari in Via Muroni n.25, codice fiscale 00196350904, rappresentato amministrativamente dal suo Direttore Prof. Marco Apollonio;

SI CONVIENE E STIPULA QUANTO SEGUE:

ART. 1

Provincia, ATC3 e Dipartimento, visto il generale e prevalente interesse scientifico della ricerca, intendono proseguire congiuntamente per il quinquennio 2009-2013, gli studi fatti fino ad oggi sulla popolazione lupo presente nei principali complessi forestali e montani della Provincia di Arezzo, con particolare riferimento all'Alpe di Catenaia con le seguenti finalità:

a) Monitorare la presenza del lupo nelle diverse aree del comprensorio;

b) Determinare la consistenza minima della popolazione su base annuale ;
c) Verificare la presenza di attività riproduttiva su base annuale;
d) Valutare la struttura e la dinamica della popolazione,
e) Valutare il livello di imparentamento dei branchi;
f) Monitorare il processo di colonizzazione di nuove aree;
g) Valutare mortalità e dispersione di soggetti di lupo mediante tecniche radiotelemetriche;
h) Approfondire le relazioni tra lupo ed ungulati selvatici nel complesso dell'alpe di Catenaia.

I dati ottenuti permetteranno di tracciare un quadro generale sulla popolazione di lupi presente nel territorio provinciale con riferimento al numero, struttura e stabilità dei branchi esistenti, ai loro rapporti di parentela, alla stabilità della loro presenza nelle diverse aree, all'entità dei processi di colonizzazione di nuove aree.
Le tecniche telemetriche e lo studio intensivo nel complesso dell'Alpe di Catenaia forniranno dati più precisi sui fattori che regolano la dinamica di popolazione del lupo, sui suoi spostamenti, sull'impatto sulle popolazioni di ungulati selvatici e domestici.
Tali informazioni costituiranno l'indispensabile supporto per la definizione delle linee di gestione che l'amministrazione provinciale di Arezzo dovrà sviluppare nei prossimi anni riguardo a due cruciali aspetti:

1) Gestione delle Oasi di Protezione; le Oasi di protezione costituiscono uno degli obiettivi prioritari della programmazione faunistica della provincia di Arezzo. Il successo nella loro gestione futura dipenderà dalla capacità di prevedere e risolvere alcune problematiche che potranno essere connesse alla loro costituzione. Tra queste si può includere la dinamica delle popolazioni di ungulati, in particolare del cinghiale, ed il contenimento, entro livelli accettabili, dei danni causati dal lupo alle attività zootecniche. I risultati alla ricerca potranno suggerire soluzioni a tali problematiche ed integrazioni all'attività di pianificazione faunistico-venatoria fino ad oggi attuata dalla Provincia di Arezzo.
2) Protezione e conservazione del lupo in Provincia di Arezzo; la ricerca fornirà un supporto indispensabile per valutare l'entità delle perdite di soggetti di lupo dovute ai vari fattori di mortalità accidentale o illegale ed il loro impatto sulla conservazione a medio termine della popolazione di lupo che gravita nel territorio provinciale.

La ricerca verrà condotta attraverso l'utilizzo dei seguenti metodi:
Wolf-howling e analisi spettrografica dei sonogrammi ottenuti dalla registrazione delle risposte;
tracciatura su neve dei branchi individuati;
analisi genetica non invasiva del DNA nucleare;
cattura e monitoraggio radiotelemetrico di alcuni soggetti di lupo.

Art. 2

PER LA REALIZZAZIONE DEL PROGETTO

La Provincia si impegna a:

- finanziare con apposito atto tra Provincia e Dipartimento le spese di base necessarie per lo svolgimento della ricerca sotto forma di contributo di ricerca, per un importo annuale di € 17.000,00 per anni cinque del progetto;
- mettere a disposizione i dati in proprio possesso relativi alle presenze faunistiche all'interno delle oasi e nelle aree ad esse limitrofe, utili ai fini della ricerca;
- provvedere alla conservazione dei campioni biologici fino al momento del loro definitivo trasferimento al Dipartimento.

L'ATC3 si impegna a:

- finanziare con apposito atto tra ATC3 e Dipartimento le spese di base necessarie per lo svolgimento della ricerca sotto forma di contributo di ricerca per un importo annuale di € 3.000,00 per anni cinque del progetto.

Il Dipartimento si impegna a:

- organizzare e partecipare alle operazioni di wolf-howling;
- organizzare e partecipare alle operazioni di tracciatura sulla neve;
- organizzare e contribuire alla raccolta di campioni biologici;

- organizzare e realizzare le operazioni di cattura di alcuni soggetti di lupo;
- elaborare i dati ottenuti mediante le tecniche sopra descritte e redigere al termine di ogni anno una relazione sullo stato di avanzamento del progetto comprensiva dei risultati conseguiti relativamente a ciascun aspetto oggetto della ricerca. Al termine di ogni anno redigere la relazione conclusiva. I contenuti delle relazioni (annuali e finale) e la loro modalità di esposizione (cartografie, figure, etc.) saranno preventivamente concordati tra i responsabili degli Enti contraenti.

Art. 3
COORDINAMENTO

Il coordinamento e le responsabilità per gli Enti vengono rispettivamente assegnati a:

- per la Provincia di Arezzo: Dott. Gabriele Chianucci – Segretario Generale della Provincia di Arezzo;
- per l'ATC3 : Sig. Giorgio Kwiatkoski – Presidente dell'Ambito Territoriale di Caccia AR3;
- per il Dipartimento: Prof. Marco Apollonio – Direttore del Dipartimento di Zoologia e Genetica Evoluzionistica

Ai responsabili è demandato il compito di concordare e garantire la realizzazione dei vari momenti operativi della ricerca nel rispetto dei tempi e delle finalità definite nel presente accordo.

Art. 4
RISULTATI DELLA RICERCA

La proprietà dei risultati della ricerca e della relazione conclusiva dei lavori congiunta e paritaria, è della Provincia di Arezzo, dell'ATC3 e del Dipartimento che potrà utilizzare comunque i risultati stessi ai propri fini scientifici e didattici istituzionali e di divulgazione.
Qualora uno dei contraenti si faccia promotore e/o partecipi ad esposizioni, congressi, convegni seminari e simili manifestazioni, nel corso delle quali si intenda esporre e far uso, sempre e soltanto a fini scientifici, dei risultati della presente convenzione sarà tenuto a citare la convenzione e i partners nel cui ambito è stata svolta la ricerca.

Art. 5
DISPOSIZIONI FINALI

Tutte le eventuali spese di registrazione sono a carico dell'amministrazione provinciale di Arezzo.

10 AGO. 2009

- Dott. Gabriele Chianucci
  Segretario Generale della Provincia di Arezzo ____________________

- Sig. Giorgio Kwiatkoski
  Presidente dell'Ambito Territoriale di Caccia AR 3 ____________________

  AMBITO TERRITORIALE DI CACCIA ATC 3 AREZZO

- Prof. Marco Apollonio
  Direttore del Dipartimento di Zoologia e Genetica Evoluzionistica dell'Università di Sassari ____________________
